# Unique and Universal Features of Epsilonproteobacterial Origins of Chromosome Replication and DnaA-DnaA Box Interactions

**DOI:** 10.3389/fmicb.2016.01555

**Published:** 2016-09-30

**Authors:** Pawel Jaworski, Rafal Donczew, Thorsten Mielke, Marcel Thiel, Stanislaw Oldziej, Christoph Weigel, Anna Zawilak-Pawlik

**Affiliations:** ^1^Department of Microbiology, Hirszfeld Institute of Immunology and Experimental Therapy, Polish Academy of SciencesWrocław, Poland; ^2^Max Planck Institute for Molecular GeneticsBerlin, Germany; ^3^Laboratory of Biopolymers Structure, Intercollegiate Faculty of Biotechnology, University of Gdańsk and Medical University of GdańskGdańsk, Poland; ^4^Department of Life Science Engineering, Fachbereich 2, HTW BerlinBerlin, Germany

**Keywords:** Epsilonproteobacteria, initiation of chromosome replication, *oriC*, DnaA, DnaA box, orisome

## Abstract

In bacteria, chromosome replication is initiated by the interaction of the initiator protein DnaA with a defined region of a chromosome at which DNA replication starts (*oriC*). While DnaA proteins share significant homology regardless of phylogeny, *oriC* regions exhibit more variable structures. The general architecture of *oriC*s is universal, i.e., they are composed of a cluster of DnaA binding sites, a DNA-unwinding element, and sequences that bind regulatory proteins. However, detailed structures of *oriC*s are shared by related species while being significantly different in unrelated bacteria. In this work, we characterized Epsilonproteobacterial *oriC* regions. *Helicobacter pylori* was the only species of the class for which *oriC* was characterized. A few unique features were found such as bipartite *oriC* structure, not encountered in any other Gram-negative species, and topology-sensitive DnaA-DNA interactions, which have not been found in any other bacterium. These unusual *H. pylori oriC* features raised questions of whether *oriC* structure and DnaA-DNA interactions are unique to this bacterium or whether they are common to related species. By *in silico* and *in vitro* analyses we identified putative *oriC*s in three Epsilonproteobacterial species: pathogenic *Arcobacter butzleri*, symbiotic *Wolinella succinogenes*, and free-living *Sulfurimonas denitrificans*. We propose that *oriC*s typically co-localize with *ruvC-dnaA-dnaN* in Epsilonproteobacteria, with the exception of Helicobacteriaceae species. The clusters of DnaA boxes localize upstream (*oriC1*) and downstream (*oriC2*) of *dnaA*, and they likely constitute bipartite origins. In all cases, DNA unwinding was shown to occur in *oriC2*. Unlike the DnaA box pattern, which is not conserved in Epsilonproteobacterial *oriC*s, the consensus DnaA box sequences and the mode of DnaA-DnaA box interactions are common to the class. We propose that the typical Epsilonproteobacterial DnaA box consists of the core nucleotide sequence 5′-TTCAC-3′ (4–8 nt), which, together with the significant changes in the DNA-binding motif of corresponding DnaAs, determines the unique molecular mechanism of DnaA-DNA interaction. Our results will facilitate identification of *oriC*s and subsequent identification of factors which regulate chromosome replication in other Epsilonproteobacteria. Since replication is controlled at the initiation step, it will help to better characterize life cycles of these species, many of which are considered as emerging pathogens.

## Introduction

Chromosome replication is tightly controlled and strictly dependent on cell cycle progression. It is primarily regulated at the first step, initiation (Zakrzewska-Czerwińska et al., 2007; Katayama et al., 2010; Skarstad and Katayama, 2013; Leonard and Grimwade, 2015). The basic mechanism of initiation is conserved in nearly all bacteria. First, the initiator protein DnaA recognizes and binds to a specific chromosomal region, the replication origin *oriC* (Ozaki and Katayama, 2009; Katayama et al., 2010; Duderstadt et al., 2011; Kaguni, 2011). This interaction leads to the formation of a highly ordered nucleoprotein complex (orisome) followed by DNA strand separation within a DNA unwinding element (DUE; Rozgaja et al., 2011; Ozaki et al., 2012; Duderstadt and Berger, 2013). The unwound DNA region provides the entry site for the assembly of a multiprotein apparatus (replisome) that synthesizes the nascent DNA strands (Beattie and Reyes-Lamothe, 2015). Most of the information on bacterial chromosome replication comes from studies in *Escherichia coli*, whose *oriC*, DnaA, and DnaA-DNA reciprocal interactions as well as the accessory and regulatory factors have been thoroughly characterized (reviewed in Katayama et al., 2010; Kaguni, 2011; Leonard and Grimwade, 2015). The initiation of chromosome replication has also been studied in a few other species (*Bacillus subtilis, Caulobacter crescentus, Mycobacterium tuberculosis, Streptomyces coelicolor*, and *Helicobacter pylori*). Comprehensive studies on these species as well as species related to *E. coli, B. subtilis, C. crescentu*s, or *Mycoplasma* sp. (Harding et al., 1982; Lartigue et al., 2003; Shaheen et al., 2009; Briggs et al., 2012) suggest that the specific activities of DnaA proteins (Zawilak-Pawlik et al., 2005), structures of the *oriC* regions (Briggs et al., 2012; Rajewska et al., 2012), modes of orisome assembly (Zawilak-Pawlik et al., 2005; Madiraju et al., 2006; Ozaki and Katayama, 2011; Briggs et al., 2012; Scholefield et al., 2012; Donczew et al., 2014), accessory proteins and regulatory mechanisms are shared by related species while being significantly different in unrelated bacteria (Wolański et al., 2014). However, it should be noted that in most bacterial species the mechanistic details of orisome assembly are still largely unknown.

*OriC*s are usually located in the vicinity of the *dnaA* and *dnaN* genes, and they can be mono- or bi-partite (Wolański et al., 2014). *OriC* regions are composed of three functional modules: a cluster (or clusters) of DnaA binding sites (DnaA boxes), a DNA-unwinding element (DUE), and sequences that bind regulatory proteins. Typical DnaA boxes are 9-mers with sequences similar to the “perfect,” high-affinity R-type *E. coli* DnaA box TTATCCACA with some degree of degeneracy (allowed mismatches ≤ 2; Wolański et al., 2014). However, different classes of “imperfect” DnaA boxes (I sites and tau boxes in *E. coli*, W-boxes in *C. crescentus*), which differ in sequence and length from the “perfect” boxes, have been shown to play important roles in DnaA oligomer assembly (McGarry et al., 2004; Kawakami et al., 2005; Ozaki and Katayama, 2009; Taylor et al., 2011). The arrangement of DnaA boxes in *oriC* (number, spacing, orientation) is not stochastic. DnaA boxes provide a molecular scaffold for sequential DnaA binding and oligomerisation, which leads to DNA unwinding in the DUE region. However, there is no “perfect” or “model” scaffold. There are a variety of DnaA box arrangements in bacterial *oriC*s, and this phenomenon is still not explained in terms of structure or function (Wolański et al., 2014; Leonard and Grimwade, 2015). The second important module, the DUE, is located outside of the DnaA box cluster, adjacent (~2 helical turns) to the last DnaA box in the scaffold. The DUE region usually contains tens of base pairs (bps) and is rich in thymines and adenines (an AT-rich region), which lower the thermodynamic stability of the DUE compared to sequences of equal AT/GC or high GC content. It has been recently shown that the region of the DUE proximal to the DnaA-box encodes a motif, a DnaA-trio, required by *B. subtilis* DnaA to open DNA and to assemble on ssDNA (Richardson et al., 2016). The last *oriC* module, the sequences that bind regulatory proteins (oriBPs, origin binding proteins), is the most divergent of all three modules (Wolański et al., 2014; Marczynski et al., 2015). These sequences can overlap with DnaA boxes or be located within the DUE or elsewhere within *oriC*. They bind different classes of proteins, such as nucleoid associated proteins (NAPs) or response regulators of two component systems. Their primary role is to efficiently transmit feedback information (positive or negative) from the environment and/or the cell itself to the *oriC* to rapidly adjust the replication rate.

Our previous work on *H. pylori oriC* revealed that it is, unlike origins of most Gram-negative bacteria, composed of two DnaA box clusters (DnaA box consensus sequence TCATTCACN), *oriC1* and *oriC2*, flanking the *dnaA* gene (Donczew et al., 2012). The DnaA protein binds to both *oriC1* and *oriC2*, bridging them together and looping out *dnaA*, in which it resembles *B. subtilis* orisome (Krause et al., 1997). Surprisingly, *oriC2*–DnaA interaction was shown to depend on DNA topology, and we identified two DnaA boxes (ts1 and ts2) which were bound only in a supercoiled form (Donczew et al., 2014). The DNA-unwinding element region is located in the *oriC2* sub-region downstream of *dnaA*. These unusual *H. pylori oriC* features raised questions of whether they are unique to this bacterium or they are also common to related species. Thus, this work was undertaken to identify and characterize *oriC* regions in bacterial species from selected Epsilonproteobacteria. Epsilonproteobacteria are found globally and inhabit a wide variety of ecological niches (Eppinger et al., 2004; Gupta, 2006). Two species of Epsilonproteobacteria, *H. pylori*, and *Campylobacter jejuni*, are undisputed human pathogens (Atherton, 2006; Epps et al., 2013). Others are proposed to be emerging pathogens connected with gastrointestinal diseases and/or reproductive disorders in animals (*Helicobacter* sp., *Campylobacter* sp., *Arcobacter* sp.). However, many Epsilonproteobacteria are non-pathogenic (symbiotic or free living species), recognized as an ecologically significant group of bacteria occurring dominantly in various redoxclines such as in deep-sea hydrothermal environments or oil fields (Nakagawa and Takaki, 2009). Such diverse life styles of Epsilonproteobacteria might be reflected by the diversity of the initiation or regulatory factors involved in the initiation of chromosome replication of the species inhabiting various ecological niches. Thus, to perform a reliable and comprehensive comparative analysis of Epsilonproteobacterial origins of chromosome replication and to compare it with *H. pylori oriC* it was reasonable to select species representing both *H. pylori*-related as well as relatively unrelated genera and lifestyles. By a two-step approach (*in silico* analysis followed by experimental *in vitro* work) we were able to precisely determine the position of *oriC* on chromosomes of pathogenic *A. butzleri*, commensal *Wolinella succinogenes* and free-living *S. denitrificans* and characterize the two most conserved modules of their *oriC* regions, namely the DnaA box clusters and the DUE. The *in vitro* bound clusters of DnaA boxes are located upstream (*oriC1*) and downstream (*oriC2*) of *dnaA*. Thus, the identified origins likely constitute bipartite origins as in *H. pylori*. The DNA-unwinding element region is located in the *oriC2* sub-region downstream of *dnaA*. The detailed comparative analysis allowed us to propose Epsilonproteobacterial *oriC* features which are typical for many origins of unrelated bacteria as well as unique for this class.

## Materials and methods

### *In silico* origin predictions

The prediction of *oriC*-type replication origins in the genomes of *A. butzleri* RM4018 [GenBank entry CP000361.1], *S. denitrificans* DSM 1251 [GenBank entry CP000153.1.1], *W. succinogenes* DSM 1740 [GenBank entry BX571656.1] was performed in a stepwise procedure, similarly as described previously (Donczew et al., 2012). Briefly, it combined GC-skew analysis, prediction of superhelicity-dependent helically unstable DNA stretches (SIDDs) in intergenic regions in the vicinity of the inflection point (minimum) of the GC-skew, and DnaA box prediction. Details are described in Supplementary Materials.

### Comparative analysis of DnaA amino acid sequences

Amino acids sequences of DnaA proteins from Proteobacteria and Actinobacteria were retrieved from UniProt amino acids sequence database (Boutet et al., 2016). To avoid sequence repetition, the search was performed on Ref90 subdatabase (Suzek et al., 2007). The Ref90 UniProt database was searched for term “chromosomal replication initiator protein DnaA” and results were further filtered to obtain sequences from Proteobacteria or Actinobacteria. Sequences of Proteobacterial DnaA proteins were further divided according to classification in the UniProt database into subfamilies: Alphaproteobacteria, Betaproteobacteria, Gammaproteobacteria, and Delta/Epsilonproteobacteria according to classification proposed by Woese (1987). All amino acid sequences were subjected to multiple sequence alignment (MSA) using MAFFT algorithm (Katoh and Standley, 2013). MSAs were performed on the whole set of sequences, as well as on subsets related to Proteobacterial subfamilies. Analysis and visualization of the MSA results were performed using BioEdit software (http://www.mbio.ncsu.edu/BioEdit/bioedit.html).

### Materials and culture conditions

The strains, plasmids and proteins used in this work are listed in Table S1. The primer sequences used in this study are listed in Table S2. The genomic DNA of *A. butzleri* RM4018, *S. denitrificans* DSM 1251, and *W. succinogenes* DSM 1740 were used as templates to amplify DNA fragments for cloning. *E. coli* was grown at 30 or 37°C on solid or in liquid Luria-Bertani medium supplemented with 100 μg/ml ampicillin or 50 μg/ml kanamycin when necessary. Plasmids and DNA fragments were purified using a GeneJET Gel Extraction Kit, GeneJET Plasmid Miniprep Kit, GeneJET Plasmid Midiprep Kit (Thermo Scientific), or Plasmid Midi AX (A&A Biotechnology). DnaA proteins were purified as described in (Zawilak-Pawlik et al., 2006) with minor modifications (Supplementary Materials). In all subsequent analyses DnaA was supplemented with 3 mM ATP (electron microscopy) or 5 mM ATP (footprinting and P1 nuclease assay).

### Footprinting, P1 nuclease assay, and primer extension (PE) reactions

DMS and DNaseI footprinting was performed as described previously (Sasse-Dwight and Gralla, 1991; Krause et al., 1997; Zawilak et al., 2001; Donczew et al., 2014). The P1 nuclease assay was conducted similarly as described (Donczew et al., 2012). Details are described in Supplementary Materials.

### Electron microscopy

Electron microscopy was performed as described previously (Donczew et al., 2012, 2014). Details are described in Supplementary Materials.

## Results

### *In silico* analysis identifies *oriCs* at the vicinity of *dnaA*

The *in silico* approach was similar to that previously applied to detect *oriC* in *H. pylori* (Donczew et al., 2012), namely a combination of GC-skew analysis, prediction of superhelicity-dependent helically unstable DNA stretches (SIDDs) in intergenic regions in the vicinity of the inflection point (minimum) of the GC-skew, and DnaA box prediction. We chose *E. coli* consensus DnaA box sequence [5′-TTWTNCACA allowing for 2 mismatches and 3 mismatches for closely-spaced DnaA boxes (Schaper and Messer, 1995)] in order not to bias the results by assuming that other Epsilonprotebacteria follow the *H. pylori* DnaA box consensus.

We identified putative origins of chromosome replication in three selected Epsilonproteobacterial species: *A*. *butzleri, W. succinogenes*, and *S. denitrificans* (Figure S1). In all three genomes, we obtained *oriC* predictions in the *dnaA* upstream region (Figure S2A), which we termed “*oriC1*” in analogy to the corresponding region in *H. pylori* (Donczew et al., 2012). The DoriC database predicts *oriC* at this position for *A. butzleri* RM4018 (DoriC entry ORI92240124) *W. succinogenes* DSM 1740 (DoriC entry ORI10010101) and *S. denitrificans* DSM 1251 (DoriC entry ORI10010173; Gao et al., 2013). We considered these *oriC1* regions less likely to represent the regions where DNA unwinding would occur because no particular DnaA box could be assigned at a distance of ~2 helical turns to the SIDDs in the expected orientation. In all three genomes, we also obtained significant *oriC* predictions in the *dnaA-dnaN* intergenic region (Figures S1, S2B). These regions are characterized by the presence of significant SIDDs accompanied by the clusters of DnaA boxes with a SIDD-proximal DnaA box (or two closely spaced boxes) located at a distance of ~2 helical turns from the right border of the SIDD (Figure S2B). We assumed that these regions contain the DUEs and we termed them “*oriC2*” in analogy to the corresponding region in the *H. pylori* replication origin where unwinding occurs (Donczew et al., 2012). We obtained one additional *oriC* prediction for *S. denitrificans*, which we termed “*oriC3*” (Figure S2C). Due to a lack of DnaA box pattern conservation, we considered this prediction less likely than the *oriC2* regions to represent the regions where unwinding would occur.

### Epsilonproteobacterial DUEs are located in the *dnaA-dnaN* intergenic region

**Figure 1:**
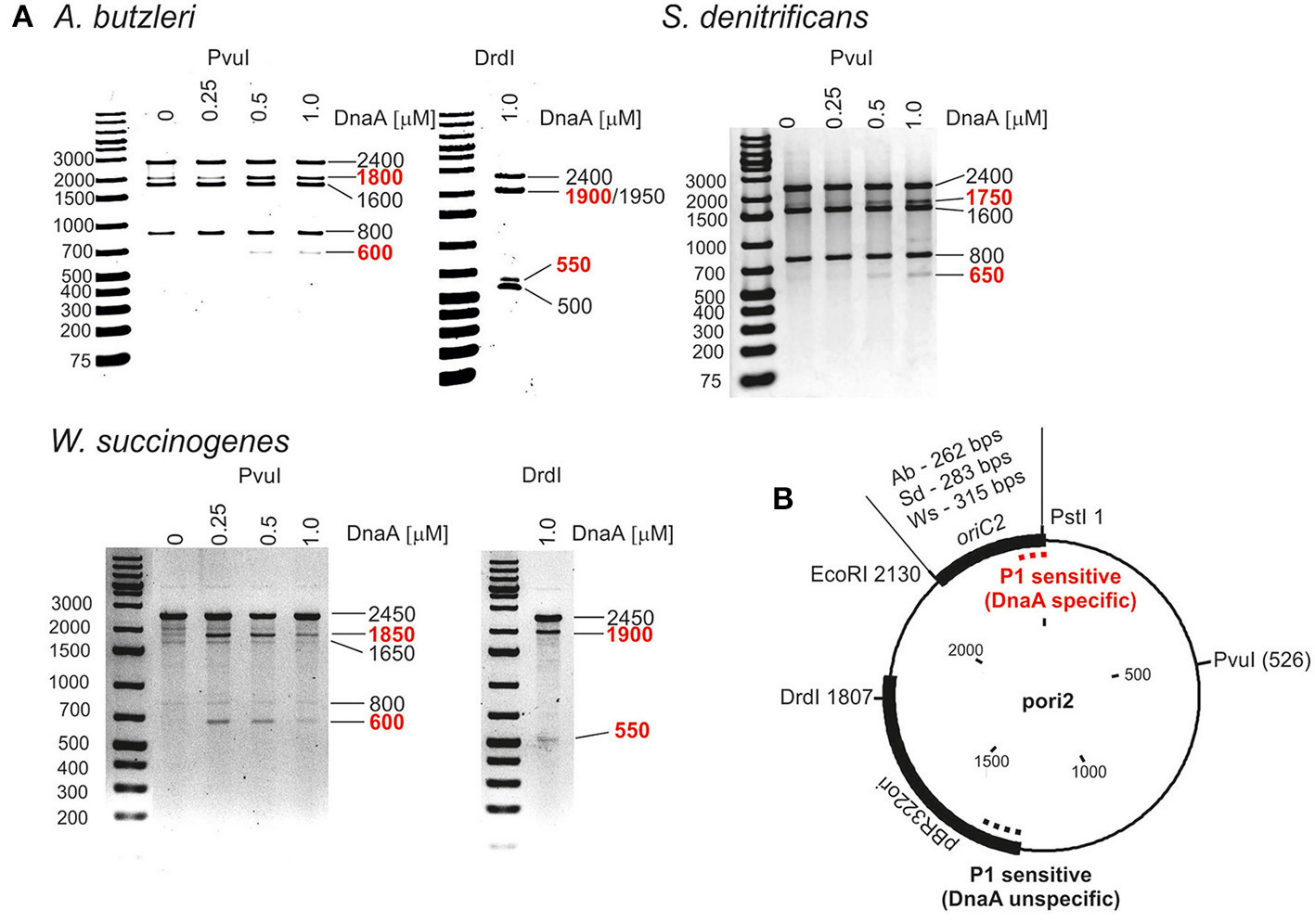
*****In vitro*** identification of DUEs in putative ***oriC*** regions of selected Epsilonproteobacteria. (A)** P1 nuclease assay determining the DNA region susceptible to DnaA-dependent unwinding. Plasmids containing putative *oriC*s with DUE regions (pAbori2, pSdori2, pWsori2) were incubated with the indicated amounts of species-specific DnaA protein, digested by P1 nuclease, and restriction digested by PvuI or DrdI. The DNA fragments were visualized by separation on 1% agarose gels and ethidium bromide staining. DNA fragments produced in a DnaA-dependent manner are marked in red. **(B)** Schematic map of the plasmids used in the assay. The most important plasmid features are marked. The specific and nonspecific P1 sensitive regions are indicated by red and black dashed lines, respectively.

Next we analyzed the putative *oriC* regions *in vitro*. Because DnaA box clusters are also found outside of the origin sites, we focused on identification of the DUE as the most reliable feature of bacterial *oriC*s (Kitagawa et al., 1998; Okumura et al., 2012; Smith and Grossman, 2015). To experimentally identify the DUE position in predicted origins, P1 nuclease assay was applied. The method is widely used to identify helically unstable regions on a DNA strand, including DUEs. For the P1 nuclease assays, a series of plasmids containing *in silico* predicted single *oriC* regions was constructed, and cognate DnaA proteins were purified (Table S1 and Figure S3). The supercoiled plasmids were incubated with increasing amounts of DnaA protein, and the resulting single-stranded DNA regions were digested with P1 nuclease. Subsequently, site-specific digestion by PvuI or DrdI excised the DNA fragment from the plasmid, the size of which allowed us to approximately estimate the position of a region unwound by DnaA. The DnaA-dependent unwinding occurred exclusively in the predicted *oriC2* regions for all analyzed Epsilonproteobacteria (Figure 1 and Figure S4). The relatively high concentration of DnaAs required for opening of the plasmid DNA at DUE suggest that there are unknown protein factors which facilitate DNA unwinding, similarly as HU enables unwinding of *E. coli oriC* (see Section Discussion). In the plasmids that were unwound in a DnaA-dependent manner (pori2 series plasmids), DNA fragments of ~550–650 bp were excised by P1/PvuI and P1/DrdI, indicating specific single-stranded DNA formation within the *oriC2* regions (Figure 1). The plasmids were also unwound at a site within the vector sequence corresponding to the plasmid origin of replication regardless of DnaA presence or concentration (all lanes contained additional DNA fragments of 800 and 500 bp in PvuI and DrdI digestion, respectively). These results are consistent with the known phenomenon that the AT-rich regions present at the origins of replication or preceding transcription units are helically unstable and may undergo spontaneous transition to a single-stranded form (Kowalski et al., 1988).

**Figure 2:**
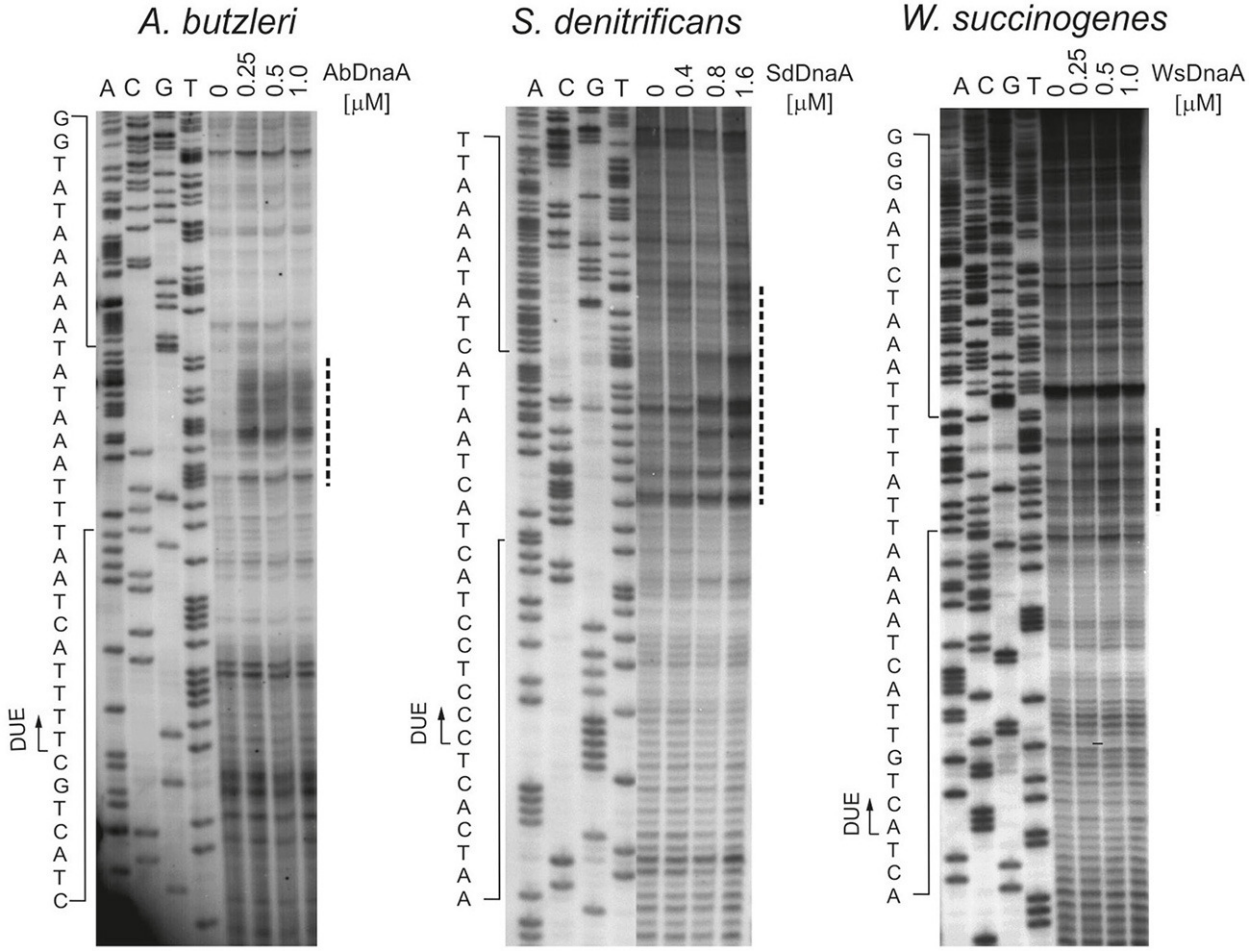
*****In vitro*** identification of the ***A. butzleri, S. denitrificans*** and ***W. succinogenes oriC*** sequence unwound by cognate DnaA proteins**. Plasmids: pAbori2, pSdori2, and pWsori2 after incubation with the indicated amounts of DnaA, were digested by P1 nuclease and used as templates for PE reactions with ^32^P-labeled primers P2, P8, and P5, respectively. Dashed-lines indicate the nucleotides susceptible to P1 nuclease treatment. The boundaries of the DUE are marked with continuous-line arrows next to the presented sequences. Complementary results, with reverse primers are presented on Figure S5.

To precisely determine the unwound regions, PE reactions with ^32^P-labeled primers were performed on P1-digested *oriC2* plasmid templates (Figure 2 and Figure S5; the primers are specified in Table S2). The primers hybridized to the template DNA ~40–80 bp away from the *in silico* predicted DUE region and were extended by Taq polymerase until it encountered the P1 nuclease digestion site. The detailed PE analysis confirmed that all *oriC2* regions underwent DnaA-dependent unwinding. Thus, they all contained DUE sequences. The main part of each identified DUE region is an AT-rich region, which is a typical feature of bacterial origins (Figure 2 and Figure S5). In *A. butzleri*, it encompasses ~26 bps and contains ~4% GC residues (overall chromosomal GC content is 27.05%). The *S. denitrificans* DUE is 40 bps long and contains 20% GC (overall 34.46%). The *W. succinogenes* AT-rich region is 28 bps long and contains 14% GC (overall 48.46%). Analyses of the DUE sequences did not detect any repeats similar to 13-mer *E. coli* L, M, R repeats in the identified AT-rich regions.

### DnaA box clusters are located upstream and downstream of the *dnaA* gene

The initial unwinding of DNA at the DUE site strictly depends on the DnaA interaction with *oriC*. Bacterial *oriC* regions usually contain one or two clusters of DnaA binding sites located in the vicinity of the DUE. They provide a platform for DnaA binding and proper oligomerisation, which leads to helix destabilization. Based on *E. coli* studies, the R-type DnaA box consensus sequence was proposed to be 5'-TTWTNCACA. However, the DnaA-binding sequences are variable, especially between distantly related species (Messer, 2002; Leonard and Méchali, 2013; Wolański et al., 2014). Thus, the precise determination of a DnaA box sequence in new species requires detailed *in vitro* analyses of the DnaA-DNA interaction. To determine the DnaA binding sites in the regions identified *in silico*, we used electrophoretic mobility shift assay (EMSA), electron microscopy (EM), and DMS footprinting.

**Figure 3:**
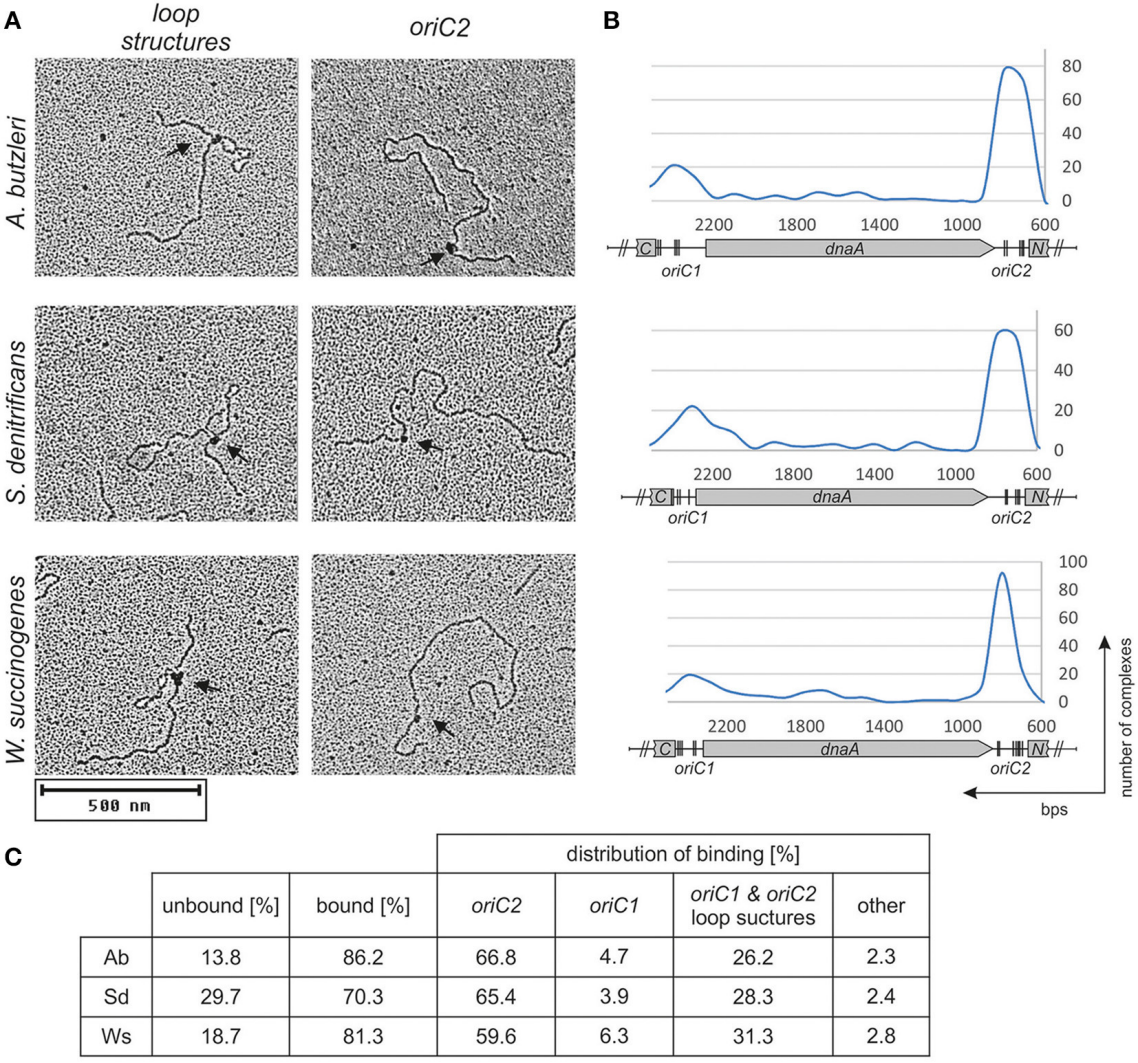
**DnaA binding to supercoiled pori1ori2 plasmids. (A)** Representative images from an EM analysis of DnaA interaction with the indicated plasmids are presented; only loop structures and DnaA-*oriC2* complexes are shown. **(B)** Histograms of complexes of supercoiled *oriC*-plasmids with cognate DnaA proteins. Distribution of complexes were calculated based on an analysis of 200 molecules for each plasmid. The most characteristic features of each plasmid are shown below the histogram: *oriC1, oriC2, dnaA*, and fragments of *ruvC* (C) and *dnaN* (N). *In silico* predicted DnaA boxes (Figure S2) are indicated by vertical bars. **(C)** Statistical distribution and the level of DnaA binding is presented in the table below micrographs. The percentage of bound molecules and distribution of complexes were calculated based on an analysis of 200 molecules for each plasmid.

Preliminary identification of DNA regions interacting with the DnaA protein was conducted using EMSA as described previously (Donczew et al., 2015). Fluorescently labeled PCR-amplified *oriC* sub-regions of *A. butzleri, S. denitrificans*, and *W. succinogenes* were incubated with increasing DnaA concentrations and subsequently resolved on a polyacrylamide gel (Figure S6). The EMSA indicated that in all analyzed origins, the DnaA protein was bound to *oriC1* and *oriC2* sub-regions; no binding of DnaA to the putative *S. denitrificans oriC3* sub-region was observed. The binding of DnaA to *oriC1* and *oriC2* sub-regions was confirmed by electron microscopy (Figure 3). The pAbori1ori2, pSdori1ori2, and pWsoriori2 plasmids, containing *oriC1* and *oriC2* sub-regions separated by a *dnaA* gene, were incubated with corresponding DnaA proteins. The nucleoprotein complexes were subsequently stabilized by glutaraldehyde crosslinking and digested by ScaI to linearize plasmid molecules. The analysis revealed that the majority (70–90%) of the analyzed plasmid molecules were bound by DnaA (Figure 3). The incubation of DnaA with supercoiled plasmids led to formation of two predominant kinds of nucleoprotein complexes: 1/looped DNA structures (~25–30% of all bound molecules) with a single protein complex bound to two distant DNA regions (Figures 3A,C). The distance measurements between the plasmid ends and the protein core on ScaI digested nucleoprotein complexes confirmed the simultaneous binding of DnaA to *oriC1* and *oriC2* (Figures 3B,C); 2/plasmid molecules with a single protein complex bound to a single plasmid region, which constituted ~68–74% of all bound molecules (Figures 3A,C). The distance measurements confirmed the binding of DnaA to *oriC1* or *oriC2*. 60–67% of the molecules were bound at *oriC2* while 4–6% of the molecules were bound at *oriC1*. Approximately 3% of all the plasmid molecules were bound at unspecific regions (Figure 3C). This analysis suggested that DnaA exhibits higher affinity toward *oriC2* than toward *oriC1* or that the complexes formed at *oriC2* are more stable than those formed on *oriC1*. The interaction between DnaA molecules bound to two suborigins apparently stabilized DnaA interactions with *oriC1*, because the majority of all *oriC1* regions which were bound by DnaA were simultaneously joined to a protein complex interacting with *oriC2*. In summary, EMSA and EM confirmed DnaA binding to *oriC1* and *oriC2* and suggested that the origin organizations in *A. butzleri, S. denitrificans*, and *W. succinogenes* resembled that of *H. pylori* (Donczew et al., 2012).

**Figure 4:**
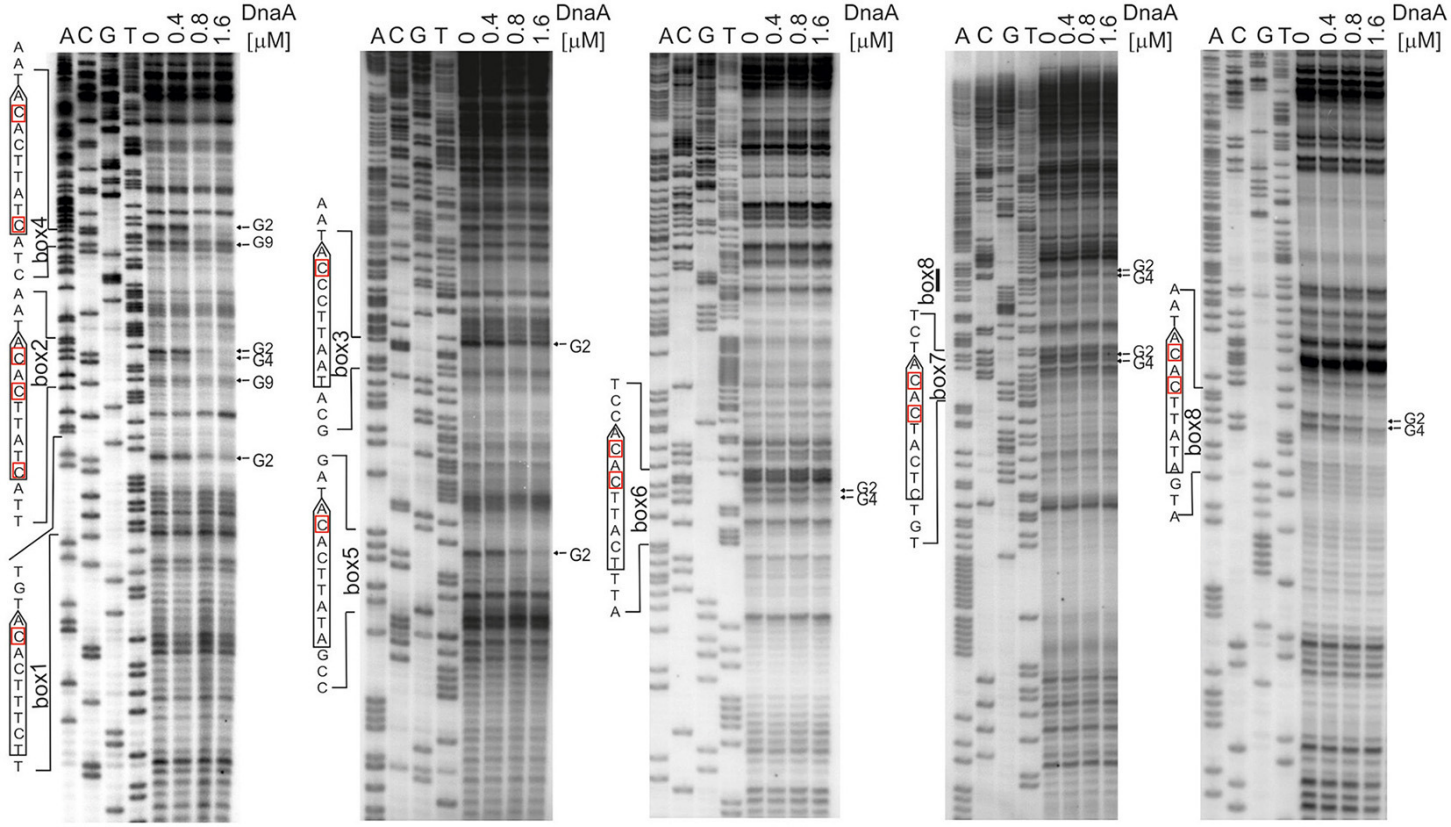
**Identification of the DnaA boxes in the ***S. denitrificans oriC*** region. DMS footprinting analysis of DnaA-***oriC*** interactions**. Plasmid pSdori1ori2 was incubated with the indicated concentrations of the DnaA protein, methylated with DMS and used as a template for PE reactions; primers used to map DnaA boxes are specified in Table S2. Sequences of identified boxes are presented on the left of each panel; protected guanosine residues (G) are indicated with arrows. Densitometric plots are presented on Figure S7. Similar experiments were conducted for *A. butzleri* and *W. succinogenes* (Figures S8, S9, respectively).

**Figure 5:**
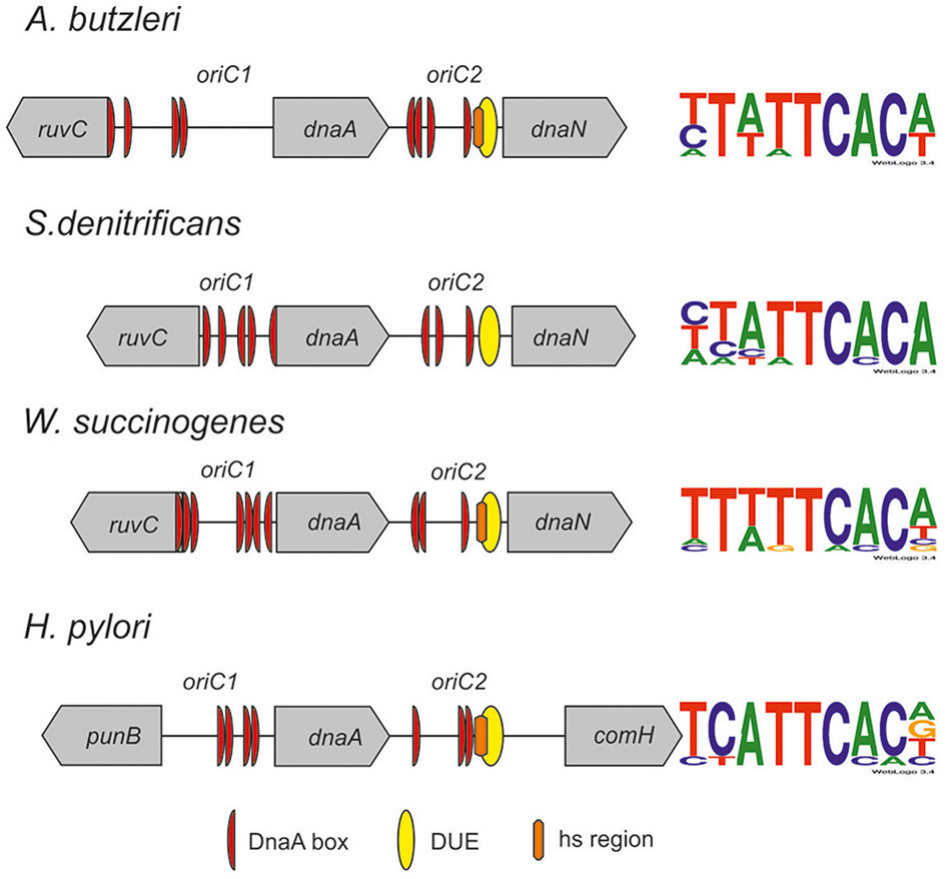
**Schematic presentation of the most characteristic features identified by DMS and P1 nuclease assay in origins of replication of selected Epsilonproteobacteria**. DnaA boxes, DUE and DMS hypermethylated regions (hs regions) similar to those identified by Donczew et al., 2014 in *H. pylori* are presented. The picture is not drawn to scale. The consensus Epsilonproteobacteria DnaA box sequences are based on the DMS analyses presented in this work and by Donczew et al., 2014. The logos were made with the WebLogo website (http://weblogo.threeplusone.com/). Detailed sequences of *S. denitrificans, A. butzleri*, and *W. succinogenes oriC* regions are presented on Figures S7–S9, respectively.

The next step was to identify DnaA boxes at *oriC1* and *oriC2* by DMS footprinting. The DMS footprinting method is based on the specific methylation of guanine and, to a lesser degree, adenine residues by dimethyl sulfate. As a result of methylation, the proximate phosphodiester bond of the DNA backbone becomes susceptible to piperidine cleavage. Proteins bound to specific DNA regions hinder DMS modification and, consequently, nucleic acid fragmentation. A subsequent primer extension reaction allows the identification of the protein binding site, which becomes apparent as decreased intensity of DNA bands on a footprinting gel. Plasmids (Table S1) containing the investigated sub-regions of *A. butzleri* (pAbori1 and pAbori2), *S. denitrificans* (pSdori1 and pSdori2), and *W. succinogenes* (pWsori1 and pWsori2) were incubated with increasing concentrations of cognate DnaA protein, methylated by DMS and piperidine-cleaved. To determine protein binding sites, sets of primers that were complementary to the upstream regions of putative DnaA boxes (Table S2) were used in the PE reactions. We detected multiple G residues protected by DnaA protein in both origin sub-regions of *S. denitrificans, A. butzleri*, and *W. succinogenes* (Figure 4 and Figures S7–S9). The subsequent comparison of the DNA sequences in the vicinity of protected G residues identified 8–10 DnaA boxes at the *oriC1*-*dnaA*-*oriC2* regions for each of the investigated Epsilonproteobacteria (Figure 5 and Figures S7–S9). It should be noted that although the DnaA concentrations required for detection of DnaA-DNA interactions were relatively high (between 0.4 and 1.6 μM), the specificity of the binding was maintained, because only G residues located within the sequence similar to *E. coli* 5′TTWTNCACA motif were protected from DMS modification. Other G residues, present elsewhere in the region, with the exception of a region that becomes hyper-methylated upon DnaA binding to *oriC2* in *A. butzleri* and *W. succinogenes* (hs region, Donczew et al., 2014), were insensitive to DMS treatment (see Section Discussion). The exact localization of DnaA binding sites, orientation and number of boxes in *oriC1* differs greatly among selected Epsilonproteobacteria (Figure 5 and Figures S7B, S8C, S9C), whereas the *oriC2* region preserved the general features of a typical bacterial origin of replication. These features include the distance between the DUE and R1_*E*. *coli*_—type DnaA box (~8–18 bps, R1_*E*. *coli*_—type box is a DUE-proximal DnaA box in reverse orientation, as in the R1 box in *E. coli oriC*), the orientation of the R1_*E*. *coli*_—type box in respect to the DUE, and the opposite arrangement of the DUE-distal box. We were unable to confirm DnaA binding to the *in silico* predicted pairs of head-to-tail boxes that are essential for the formation of a functional orisome in *H. pylori* and that play a crucial role in its DUE unwinding (ts boxes; Donczew et al., 2014; see Section Discussion). The sequences of *in vitro* determined DnaA boxes were assembled to generate a consensus DnaA box sequence for each studied bacterium (Figure 5): *A*. *butzleri* 5′ HTWTTCACW, *S. denitrificans* 5′ HHATTCACA, and *W. succinogenes* 5′ TTWTTCACN. The comparison of DnaA box consensus sequences of the analyzed Epsilonproteobacteria, together with the consensus *H. pylori* DnaA box sequence 5′-TCATTCACN (Donczew et al., 2014 and Figure 5), revealed that between the species the boxes are relatively diverse in the first three and the very last nucleotide positions, similarly as was observed before in other bacteria (Tsodikov and Biswas, 2011; Wolański et al., 2014), but they are characterized by a conserved 5-nt core sequence 5′-TTCAC (4–8th residue of a 9-mer; Figure 5). We propose that this core sequence is a hallmark of DnaA boxes in Epsilonproteobacteria which distinguishes these boxes from other bacterial species.

### Epsilonproetoabcterial dnaA's specificity toward DnaA boxes is different than that of *E. coli* and *M. tuberculosis* DnaAs

Experimentally identified Epsilonproteobacterial DnaA boxes follow the general DnaA box pattern. However, we observed two distinct features of these boxes: the conserved T residue at the 5th position of the DnaA box and the protection of two G residues by DnaA from DMS modification (G residues at the bottom strand of the DnaA box). In 33 DnaA boxes identified in Epsilonproteobacteria (26 in this work and 7 in *H. pylori*) the fifth position of the DnaA box was occupied by a T residue. In contrast, in *E. coli* and *M. tuberculosis*, the C residue is preserved at the 5th position of the DnaA box. However, it should be noted that this residue is not important for sequence-specific *E. coli* and *M. tuberculosis* DnaA binding to DnaA boxes (Schaper and Messer, 1995; Fujikawa et al., 2003; Tsodikov and Biswas, 2011). All of the identified Epsilonproteobacterial DnaA boxes were protected at both G residues from DMS modification upon DnaA binding. In similar DMS experiments, *E. coli* and *M. tuberculosis* DnaA proteins protect the 2nd guanine residue (G2) while expose the 4th guanine residue (G4) (Grimwade et al., 2000; Madiraju et al., 2006; Kaur et al., 2014). These two unique features prompted us to confirm the intrinsic ability of Epsilonproteobacterial DnaAs to interact with G4 of the DnaA box and the importance of the T5 residue for Epsilonproteobacteria DnaA-DNA interactions.

**Figure 6:**
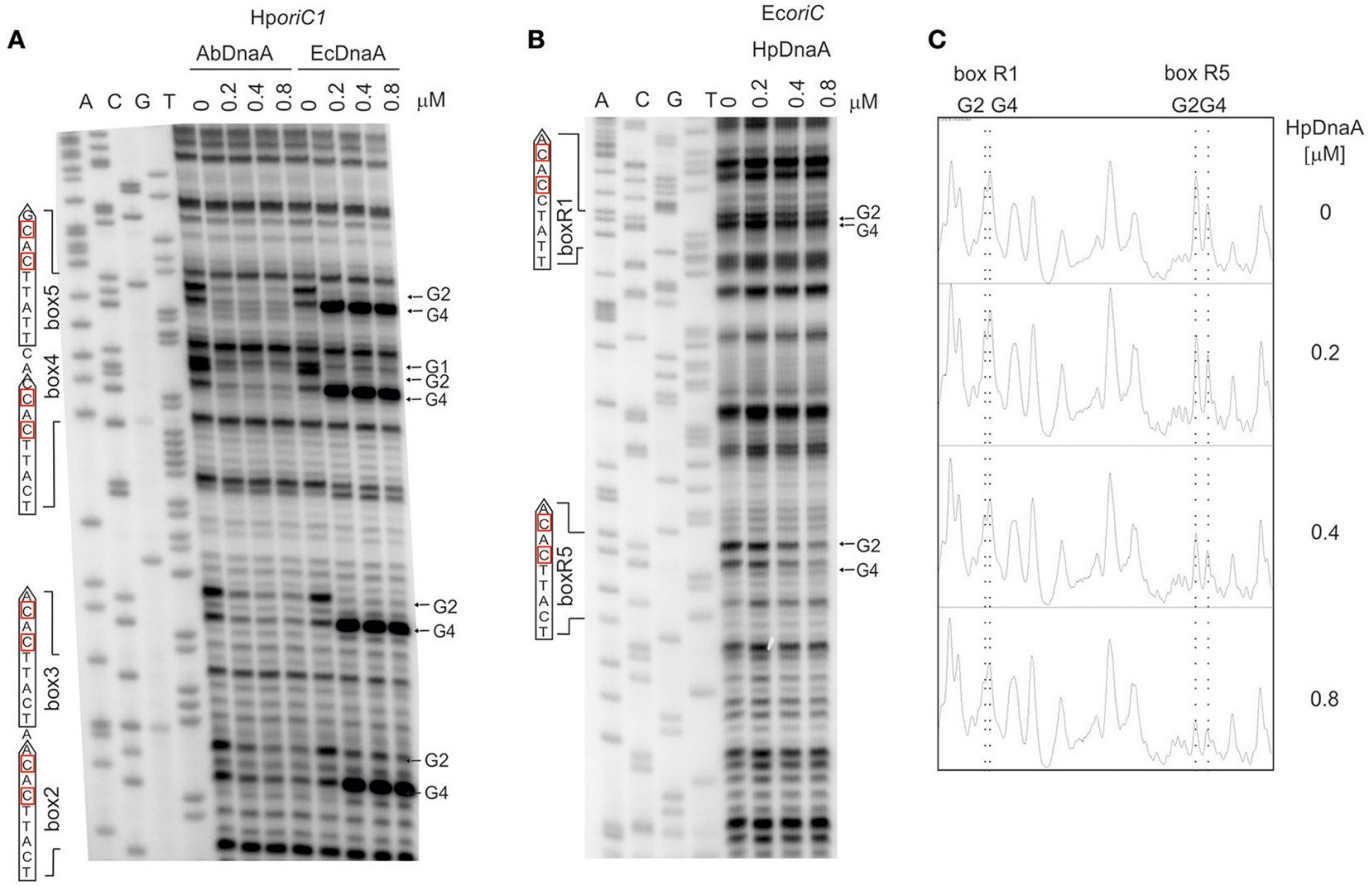
**The specificity of interaction between Epsilonproteobacterial DnaAs and DnaA boxes. (A)** DMS footprinting analysis of interactions of *A. butzleri* and *E. coli* DnaA with *H. pylori oriC1*. Plasmid pori1ori2 was incubated with the indicated DnaA protein concentrations, methylated with DMS and used as a template for PE reactions; primers used to map DnaA boxes are specified in Table S2. Sequences of identified boxes are presented on the left of each panel; protected guanosine residues (G) are indicated with arrows. **(B)** DMS footprinting analysis of interactions between *H. pylori* DnaA and *E. coli oriC*. Plasmid pOC170 was incubated with the indicated concentrations of the DnaA protein, methylated with DMS and used as a template for PE reactions; primers used to map DnaA boxes are listed in Table S2. Sequences of identified boxes are presented on the left of each panel; protected guanosine residues (G) are indicated with arrows. **(C)** Densitometric plots, which supplement the footprinting data. The plots were obtained for the lanes corresponding to the indicated amounts of DnaA protein. Protected guanosine residues (G) are indicated with dotted lines.

First, we directly compared the *E. coli* DnaA and *A. butzleri* DnaA interactions with *H. pylori oriC* regions. For both proteins the boxes were not optimal, neither in sequence of a single box nor in an overall organization of DnaA boxes in *oriC*. However, they should be recognized by both proteins since they contain the core sequence 5′-TTCAC (4–8 bps) important for Epsilonproteobacteria (represented by *A. buztleri* DnaA here), and recognizable by *E. coli* DnaA. We applied the DMS footprint assay to be able to observe interactions of DnaAs with guanines. Both proteins bound boxes located at *H. pylori oriC1* (boxes 2–4; Donczew et al., 2014; Figure 6A). Both proteins recognized *H. pylori* DnaA boxes according to their intrinsic molecular pattern—*A. butzleri* DnaA protected both G residues (G2 and G4) while *E. coli* DnaA protected G2 and exposed G4 to DMS (Figure 6A). The differences in DnaA interaction with G residues between Epsilonproteobacterial DnaAs and EcDnaA were confirmed by DNaseI footprinting. The *H. pylori* GST-HpDnaA(IV) protein (Zawilak et al., 2001) interacted with boxes 2–3 and 4–5; this interaction almost entirely protected the boxes from DNaseI digestion (Figure S10). *E. coli* DnaA also interacted with these boxes, however, in contrast to *H. pylori* DnaA, it exposed DNA to DNaseI digestion at positions corresponding to G4 of each DnaA box.

To verify the importance of the presence of T residue at 5th position of the DnaA box for the interaction with Epsilonproteobacterial DnaA we analyzed interactions of *H. pylori* DnaA with *E. coli oriC* by DMS footprinting. We were able to detect binding of *H. pylori* DnaA to the *E. coli* R5 DnaA box, which is identical to the strong *H. pylori* c2 and c3 DnaA boxes, i.e., it contains T at the 5th residue. We could also detect significantly weaker interaction of *H. pylori* DnaA with the R1 DnaA box which contains C at the 5th position (Figures 6B,C). This suggests that T residue at the 5th position in the DnaA box is important for *H. pylori* DnaA binding to DnaA boxes. Notably, *H. pylori* DnaA protected both G residues of *E. coli* DnaA boxes.

**Figure 7:**
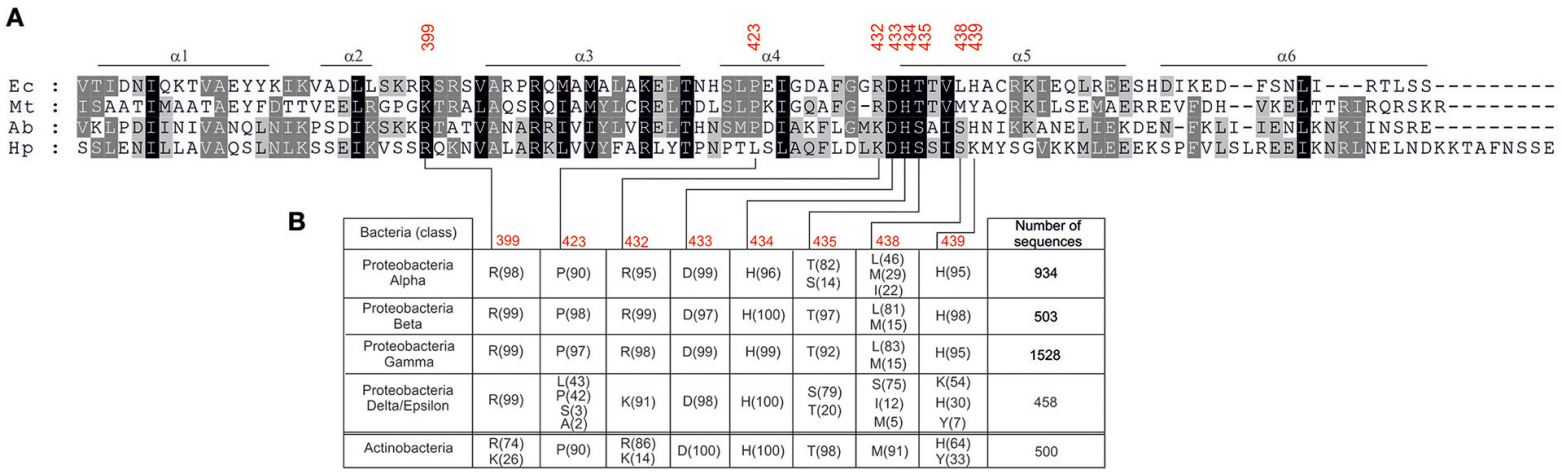
**Amino acids occurrence frequency at interface between DnaA domain IV and DNA for various bacterial classes. (A)** Multiple-sequence alignment of DnaA homologs from *E. coli* (Ec), *M. tuberculosis* (Mt), *A. butzleri* (Ab), and *H. pylori* (Hp). The red numbers indicate amino acids in *E. coli* DnaA involved in base-specific interactions. **(B)** The amino acid residues occurring most frequently in positions responsible for base-specific DNA-DnaA interactions are presented. Proteobacterial sequences are divided into classes, the numbers of analyzed sequences are shown in the last column, the frequency (in percent) of the amino acid occurrence at each position is shown in parenthesis.

Further analyses are required to reveal the molecular interactions between DnaA and DNA which could explain the observed distinctions in DnaA box recognition between Epsilonproteobacterial DnaAs and *E. coli* DnaA. It has been previously shown that base-specific interactions with major and minor grooves of the DnaA box DNA are made by amino acid residues located at three regions of domain IV of DnaA: a basic loop between helix 2 and helix 3 (residue 399 in *E. coli*), helix 4 (residue 423 in *E. coli*), and helix 5 (residues 432–435, 438–439 in *E. coli*; Blaesing et al., 2000; Fujikawa et al., 2003; Tsodikov and Biswas, 2011). As it is shown in Figure 7, the arginine equivalent to *E. coli* R399 is maintained in all of the investigated Epsilonproteobacterial sequences. The DNA-protein interaction site located on helix 4 displays some more diversity - the position equivalent to P423 in *E. coli* DnaA is occupied by proline (e.g., *A. butzleri* DnaA) or leucine (e.g., *H. pylori* DnaA) residues in Epsilonproteobacteria. However, taking into account that *A. butzleri* DnaA and *H. pylori* DnaA display similar specificity of DnaA-DNA interactions, the diversity within helix 4 is probably not responsible for the observed DnaA box recognition pattern. Thus, most of the observed changes might arise from base-specific interactions between DNA and amino acids located at the N-terminus of helix 5 of DnaA's domain IV (Figure 7). This motif is about eight residues long and begins with positively charged residues corresponding to R432 in *E. coli*. Interestingly, for most of the Proteobacterial families arginine in this position is observed, however in the case of Epsilon/Delta Proteobacteria mostly a lysine residue occurs. The next, highly conservative HD dyad (positions 433–434 in *E. coli*) is followed by a threonine residue in most of the investigated sequences (position 435 *E. coli*). However, for Epsilon/Delta Proteobacteria this position is occupied mostly by serine residues or, less frequently, by threonine or alanine (see Figure 7). The next residue, that directly interacts with DNA, is located in position 438 (in *E. coli*). That position is usually occupied by hydrophobic residues (leucine, methionine), however, for many epsilon/delta proteobacterial DnaA sequences a polar serine residue is present at position 438. The last element of the helix 5 DNA binding motif is a position equivalent to *E. coli* H439, which is maintained in most of the analyzed sequences (Figure 7). In the case of epsilon/delta proteobacteria, histidine, and tyrosine was also observed (30 and 7%, respectively), but a lysine residue was the most frequently present (54%). In conclusion, the performed analysis of DnaA domain IV amino acid sequences of Proteobacteria and Actinobacteria reveals that especially positions equivalent to *E. coli* DnaA residues 435, 438–439 display some significant variation which could be responsible for the observed differences in DnaA box recognition between Epsilonproteobacterial DnaAs and DnaAs of other bacterial classes. However, more detailed studies are required to find molecular/structural features responsible for Epsilonproteobacterial DnaAs specificity toward their DnaA boxes.

## Discussion

It has been recently proposed that bacterial *oriC* regions are central management systems controlling DNA replication as well as responsible for coordination of replication with other cellular processes (Marczynski et al., 2015). Thus, identification of the origin of chromosome replication is the first step in characterization of initiation of chromosome replication at the level of molecular mechanism as well as in the context of the cell cycle in individual bacterial species.

In this work, we identified and characterized experimentally the putative *oriC* regions of three Epsilonproteobacteria, namely *A. butzleri, S. denitrificans*, and *W. succinogenes*. We determined the overall structures of these regions as well as the sequences of individual DnaA boxes and DUEs. These analyses allowed us to propose features which are specific to the Epsilonproteobacteria as well as those which are common to bacteria in general. It should be noted that due to the lack of molecular biology techniques available for studies on the three Epsilonproteobacterial species the functionality of these origins has not been characterized *in vivo*. However, despite being “putative” origins they should be considered as reliably identified because all of the characterized modules (DnaA boxes, bipartite structure, and DUE) follow either the general or *H. pylori* specific *oriC* schemes.

### General structure of epsilonproteobacterial *oriCs*

Similarly as previously shown for most other bacteria including *H. pylori*, the putative origins of chromosome replication of *A. butzleri, S. denitrificans*, and *W. succinogenes* are located in the vicinity of *dnaA*. Two clusters of DnaA boxes flank the *dnaA* gene; the DUE is located in the *dnaA* and *dnaN* intergenic region (Figure 5). Interestingly, in all Epsilonproteobacteria, with the exception of several species of the *Helicobacter* genus, the *ruvC*-*dnaA*-*dnaN*-*gyrB* locus is highly conserved (Figure S11A). The so far presented experimental results together with the *in silico oriC* predictions conducted for a few randomly chosen species of the same class suggest that Epsilonproteobacterial origins might be bipartite, with *oriC1* and *oriC2* conservatively located between *ruvC*-*dnaA* and *dnaA*-*dnaN*, respectively (Figure 5 and Figure S11B; data for *oriC1* are not shown). The manner of DnaA interaction with the entire *oriC1-dnaA-oriC2* region resembles that presented previously for *oriCs* of *H. pylori* and *B. subtilis*, and also for the *E. coli oriC-mioC* region and *S. coelicolor oriC* (Krause et al., 1997; Jakimowicz et al., 2000; Donczew et al., 2012). As observed on electron micrographs, ~1/3 of DNA molecules adopt the looped structure, in which two *oriC* sub-regions (or DnaA-DNA subcomplexes in *E. coli* and *S. coelicolor*) are joined together by the protein-protein interactions between DnaA molecules bound to each sub-region. This indicates that the DnaA protein of these species is characterized by intrinsic ability to join DnaA-DNA subcomplexes, provided that such subcomplexes are located on the same molecule, because no specific intermolecular interactions were observed between subcomplexes located on separate plasmid molecules. The clusters of DnaA boxes can also be bound independently by Epsilonproteobacterial DnaA, with predominant binding to *oriC2*. This indicates a higher affinity of Epsilonproteobacterial DnaA toward DUE proximal *oriC2* or increased stability of DnaA-*oriC2* complexes over DnaA-*oriC1* complexes. In fact, the DnaA-*oriC1* complexes were rarely observed in Epsilonproteobacterial *oriC1-dnaA-oriC2* plasmids (Figure 3C and Donczew et al., 2014). However, as was shown for *H. pylori*, when *oriC1* is detached from *oriC1-dnaA-oriC2* context, it is efficiently bound by *H. pylori* DnaA as linear or supercoiled DNA (Donczew et al., 2012). This further supports the hypothesis of a complex interplay between *oriC1* and *oriC2* sub-regions. The role of such interplay is still not explained. For bipartite chromosomal and plasmid origins the regulatory role of such interaction is proposed (Krause et al., 1997; Moriya et al., 1999 and references herein). It is still not known what is the role of DnaA-mediated interaction between *oriC* and DnaA boxes at the *mioC* promoter in *E. coli*, but it might be related to an interplay between *oriC* activity and *mioC* transcription (Løbner-Olesen and Boye, 1992; Bates et al., 1997; Su'etsugu et al., 2003; Lies et al., 2015).

The DUEs of the three identified Epsilonproteobacterial origins, similarly as in *H. pylori* and *B. subtilis*, are located in *oriC2*. They are composed of the AT-rich sequence with no tandem repeats similar to *E. coli* L, M or R 13-mers. However, in all three species the DnaA-trio motif is found (Richardson et al., 2016). In *A. butzleri* and *W. succinogenes* a hypersensitivity to DMS of the region between R1_*E*. *coli*_—type DnaA box and DUE was observed, which resembles *H. pylori* hs region (Donczew et al., 2014). Although this phenomenon is not fully explained, it further confirms the correct assignment of Epsilonproteobacterial DUEs. Similarly to *H. pylori*, the interaction between *oriC2* and DnaA is sufficient to unwind the DUE, which suggests that *oriC1* plays additional role(s) in orisome assembly and/or regulation of chromosome replication. The *oriC1* sub-region might be particularly important *in vivo* since its deletion is lethal in *H. pylori* and *B. subtilis*. It should be noted that, although DNA unwinding was driven by a relatively high DnaA concentration, it was localized exclusively in *oriC2*, 8-18 bps downstream of the R1-type DnaA box. Thus, it can be considered as highly specific DnaA dependent unwinding. However, it can't be excluded that another protein facilitates DnaA-dependent DUE unwinding in Epsilonproteobacteria, such as *E. coli* HU is indispensable for *E. coli* DUE opening *in vitro* (Hwang and Kornberg, 1992). Further studies are required to identify such protein(s) involved in assembly and/or regulation of Epsilonproteobacterial orisomes.

By detailed DMS footprinting we identified all the boxes bound *in vitro* by DnaA at *oriC1* and *oriC2* (Figure 5). The *oriC* boxes of the three species analyzed in this work and of *H. pylori* differ in number, orientation, and consensus sequence (see also below). Thus, we did not find any particular pattern of DnaA box arrangement conserved among the four Epsilonproteobacterial species. However, the orientation of the last box in a cluster, the DUE-proximal R1_*E*. *coli*_—type box, is conserved in all four species. It should be noted that on the contrary to *in silico* predictions, this box is a single not a double-box as ts1 and ts2 DnaA boxes of *H. pylori*. However, ts DnaA boxes, especially the *H. pylori* ts1 DnaA box, are very weak, thus they can be easily missed in analyses conducted under sub-optimal conditions such as *in vitro* studies. Nonetheless the identified R1_*E*. *coli*_—type DnaA boxes are oriented toward the DUE, as in all bacterial origins characterized so far (Rajewska et al., 2012; Wolański et al., 2014). The distance between the DUE and the adjacent *in vitro* bound DnaA box varies between 8 and 18 bps and is typical for the majority of known origins (Figures S7–S9). Interestingly, in the three analyzed species but not in *H. pylori*, the DUE-distal DnaA box in *oriC2* is oriented outward from the DUE-proximal DnaA box. This feature is in agreement with other data showing that the clusters of DnaA boxes in many bacteria are characterized by similar tail-to-tail (outward) orientation of the distal boxes (Rajewska et al., 2012; Wolański et al., 2014). In *E. coli*, this orientation of the distal boxes as well as the asymmetrical orientation of left and right DnaA box clusters was proposed to be required for the formation of two oppositely-oriented DnaA subcomplexes (Rozgaja et al., 2011; Noguchi et al., 2015). The significance of this DnaA box orientation beyond the *E. coli* initiation complex is not known, but it is possible that the oppositely polarized DnaA oligomers are important for different orisome functions, such as unwinding of DNA and loading of other replisome proteins. It has been proposed that similar oppositely polarized and functionally divided DnaA oligomers might be formed on bipartite origins such as in *H. pylori*, but, taking into account the loop formation between suborigins, the uniform orientation of the boxes would be then required (Noguchi et al., 2015). However, the orientation of the DnaA boxes in three other Epsilonproteobacteria is not uniform. In all cases *oriC2* DnaA binding sites are organized in two oppositely-directed (tail to tail) arrays of boxes. The *in vitro* bound DnaA boxes in *A. butzleri oriC1* are oriented in the same direction, while DnaA boxes at *oriC1* of *W. succinogenes* and *S. denitrificans* are oppositely-directed. Interestingly, the terminal boxes at *W. succinogenes* and *S. denitrificans oriC1* are oriented inward (head-to-head) while those at *oriC2* are oriented outward. This raises interesting questions of whether polarized DnaA oligomers are formed on bipartite (sub)origins, and what is the role of individual suborigin-DnaA complexes on orisome function.

### The specificity of dnaA-dnaA box interactions in epsilonproteobacteria

In this work, we performed a detailed analysis of the DnaA boxes at *oriCs* bound *in vitro* by DnaA proteins of the analyzed Epsilonproteobacteria. By comparing the localization and orientation of DnaA boxes at *oriC* we concluded that there is no common DnaA box pattern in Epsilonproteobacteria (Figure 5). However, we noticed that the consensus sequence of Epsilonproteobacterial DnaA box is strictly conserved within the 5-nucleotide core 5′-TTCAC-3′ (4–8th position). The importance of nucleotides at other positions is species-dependent (Figure 5). Interestingly, the *S. denitrificans* DnaA box sequence is highly degenerated at the first three positions, while positions 4–9 are well-conserved. This may suggest that *S. denitrificans* boxes are not 9-mers but 6-mers. This is similar to the 5-mer W-boxes in *C. crescentus*, 7-mer boxes of *M. tuberculosis*, or 6-mer τ-sites of *E. coli oriC* (Kawakami et al., 2005; Taylor et al., 2011; Tsodikov and Biswas, 2011). Alternatively, DMS footprinting, a very sensitive method, does not discriminate between low- and high-affinity DnaA boxes thus, the identified DnaA binding sites might belong to different classes. Various classes of boxes might be partially responsible for regulating DnaA assembly during orisome formation such as ATP- and ADP-DnaA boxes in *E. coli* or G and W boxes in *C. crescentus* (Ozaki and Katayama, 2009; Taylor et al., 2011).

Nonetheless we observed two distinct features connected with the core consensus sequence of Epsilonproteobacterial DnaA boxes and DnaA-DnaA box interactions: strict conservation of thymine at the 5th position and the binding of Epsilonproteobacterial DnaA to guanine G4 of a DnaA box. So far the 5th positions of the *E. coli* consensus DnaA box (TTWTNCACA) and the *M. tuberculosis* DnaA box (YWRTCCACA) were considered to be variable without influencing the affinity toward cognate DnaAs (Schaper and Messer, 1995; Fujikawa et al., 2003; Tsodikov and Biswas, 2011). However, it should be noted that in both species, the 5th position of the DnaA box is preferentially occupied by the C residue. All other bases of the sequence, either of the upper or the lower strand, interact with DnaA, and any deviation from the most stringent TTATNCACA consensus sequence results in reduced DnaA affinity toward the less perfect boxes. Surprisingly, all Epsilonproteobacterial DnaA boxes are strictly conserved at the 5th position, which is occupied by the T residue. The other nucleotides within a core sequence are also highly conserved. The 8th position is occupied by the C residue, with the sole exception of the *H. pylori* ts1 DnaA box, in which C at the 8th position is substituted with A. The ts1 and ts2 DnaA boxes constitute a double DnaA box, which might require special sequence adjustment for proper and/or efficient DnaA binding. In addition, the ts1 DnaA box is bound with lower affinity than the ts2 box (Donczew et al., 2014), indicating that substitution at this position of a DnaA box negatively affects DnaA binding. Relatively rare C to A and *vice versa* substitutions at 6th and 7th positions, respectively, are tolerated. Other substitutions are not tolerated. For example, the DnaA box c1 (TTATAGACA), in which T5 is substituted by an A residue while C6 by a G residue, is not bound by DnaA neither in DMS nor DNaseI footprinting (data not shown) and should not be considered as a DnaA box any longer. The C residue at the 6th position of a DnaA box, which corresponds to G at the 4th position of the DnaA box in reverse orientation (for example *H. pylori* 5′-NGTGAATGA), has been shown to be protected from DMS modification upon interactions with DnaA. Such protection indicates that, in contrast to DnaAs from other phyla, the Epsilonproteobacterial DnaA proteins directly interact with this nucleotide residue. Altogether these data suggest that the molecular interactions between Epsilonproteobacterial DnaA and cognate DnaA boxes differ from those of *E. coli* and *M. tuberculosis* DnaA with cognate DnaA boxes and our preliminary analyses suggest that these differences arise from amino acid substitutions in helix 5 of domain IV of Epsilonproteobacterial DnaAs.

In summary, the identified origins of *A. butzleri, S. denitrificans*, and *W. succinogenes* are organized in a similar manner as previously characterized bacterial origins. The *in silico* and *in vitro* analyses of the origins of four bacteria from this class followed by global chromosome sequence analysis of the available Epsilonproteobacteria species allowed us to propose *oriC* features characteristic for the class, including the typical *ruvC-dnaA-dnaN* localization of *oriC* (with the exception of *Helicobacteriaceae* species), the bipartite *oriC* structure, and the core 5′-TTCAC (5–8th nucleotides of a 9-mer) consensus DnaA box sequence. We present evidence that the molecular interaction between Epsilonproteobacteria DnaA and DnaA box is significantly different from the interactions described for other bacteria, in particular *E. coli* and *M. tuberculosis*. Our comprehensive analysis of Epsilonproteobacteria opens possibilities for more precise and considerably quicker identification of origins in other bacteria of the class as well as further identification and characterization of factors involved in regulation of replication of Epsilonproteobacterial chromosomes. Taking into account that some of the known Epsilonproteobacteria are pathogenic (*Campylobacter* sp.) or are considered to be emerging pathogens (*Arcobacter* sp.) further studies on initiation of chromosome replication, the key step in the bacterial cell cycle, might help to better characterize life cycles of these species.

## Author contributions

PJ, RD, and AZ planned the experiments. PJ, RD, and AZ performed the experiments. TM provided electron microscopy facility. CW, MT, and SO planned and performed *in silico* analyses. PJ, RD, CW, and AZ analyzed data. PJ, RD, CW, SO, and AZ wrote the manuscript.

### Conflict of interest statement

The authors declare that the research was conducted in the absence of any commercial or financial relationships that could be construed as a potential conflict of interest.
