# Supplementary material for: Unique and Universal Features of Epsilonproteobacterial Origins of Chromosome Replication and DnaA-DnaA Box Interactions
Source: Front Microbiol. 2016 Sep 30;7:1555. doi: 10.3389/fmicb.2016.01555 (PMC5043019; doi:10.3389/fmicb.2016.01555)
Supplement: Supplementary file 1 [file Image1.PDF]

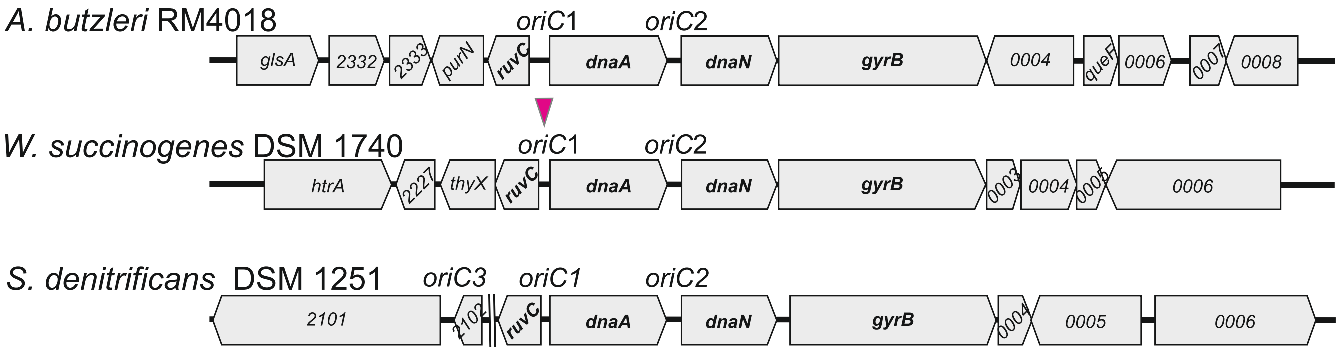

Figure S1 Schematic representation of *oriC* regions identified *in silico* in selected Epsilonproteobacteria. Genes are presented as grey boxes with gene name, and the direction of transcription is indicated by an arrowhead. Putative origins and GC-skew minima are marked above the intergenic regions by *oriC* and pink triangle, respectively.
