# Supplementary material for: Unique and Universal Features of Epsilonproteobacterial Origins of Chromosome Replication and DnaA-DnaA Box Interactions
Source: Front Microbiol. 2016 Sep 30;7:1555. doi: 10.3389/fmicb.2016.01555 (PMC5043019; doi:10.3389/fmicb.2016.01555)
Supplement: Supplementary file 2 [file Image2.PDF]

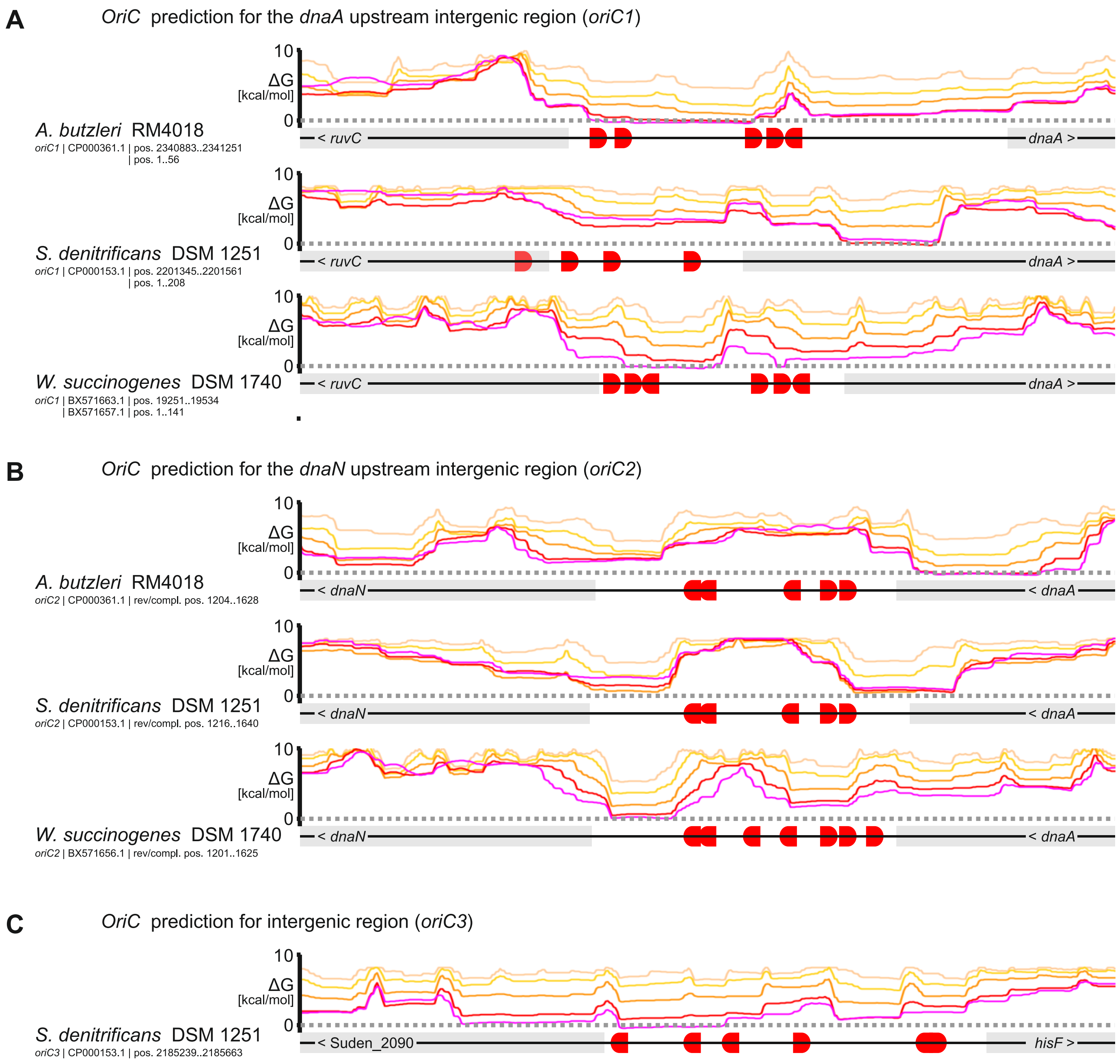

Fig. S2 SIDD plots and DnaA box assignments for *oriC* predictions for *A. butzleri* RM4018, *S. denitrificans* DSM 1251 and *W. succinogenes* DSM 1740. Y-axis = free energy values calculated for individual base pairs by WebSIDD (http://benham.genomecenter.ucdavis.edu/sibz/) for different degrees of negative superhelicity of the input sequence (pink $\sigma = -0.06$; red $\sigma = -0.055$; dark orange $\sigma = -0.05$; light orange $\sigma = -0.045$; pale orange $\sigma = -0.04$) and plotted as a graph (values ≤ 0 kcal/mol indicate strand opening). X-axis = DNA sequence by position. DnaA boxes = red, curvature indicates orientation. Flanking genes = grey boxes, and gene name and direction of transcription are indicated by arrowhead. Elements are shown to scale.
