# Supplementary material for: Unique and Universal Features of Epsilonproteobacterial Origins of Chromosome Replication and DnaA-DnaA Box Interactions
Source: Front Microbiol. 2016 Sep 30;7:1555. doi: 10.3389/fmicb.2016.01555 (PMC5043019; doi:10.3389/fmicb.2016.01555)
Supplement: Supplementary file 3 [file Image3.PDF]

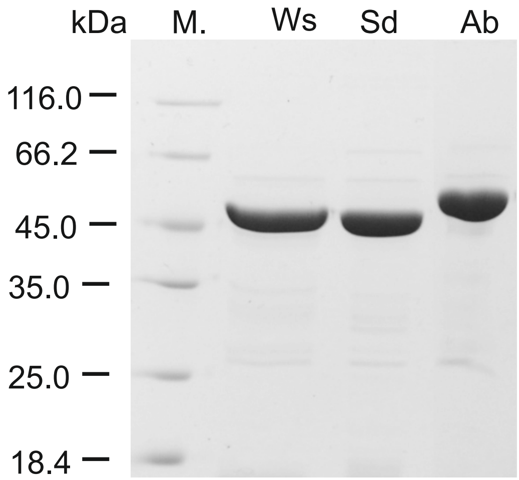

Figure S3 6HisDnaA protein purification. 6HisDnaA proteins were isolated from *E. coli* BL21. Proteins eluted with buffer containing 100 mM imidazole were analysed in a 10% SDS-PAGE gel (5 μg/well) and stained with Coomassie brilliant blue. M, molecular weight marker; Ws, *Wolinella succinogenes* 6HisDnaA (Mw 54,49 kDa); Sd, *Sulfurimonas denitrificans* 6HisDnaA (Mw 54,33 kDa); Ab, *Arcobacter butzleri* 6HisDnaA (Mw 54,48 kDa).
