# Supplementary material for: Unique and Universal Features of Epsilonproteobacterial Origins of Chromosome Replication and DnaA-DnaA Box Interactions
Source: Front Microbiol. 2016 Sep 30;7:1555. doi: 10.3389/fmicb.2016.01555 (PMC5043019; doi:10.3389/fmicb.2016.01555)
Supplement: Supplementary file 4 [file Image4.PDF]

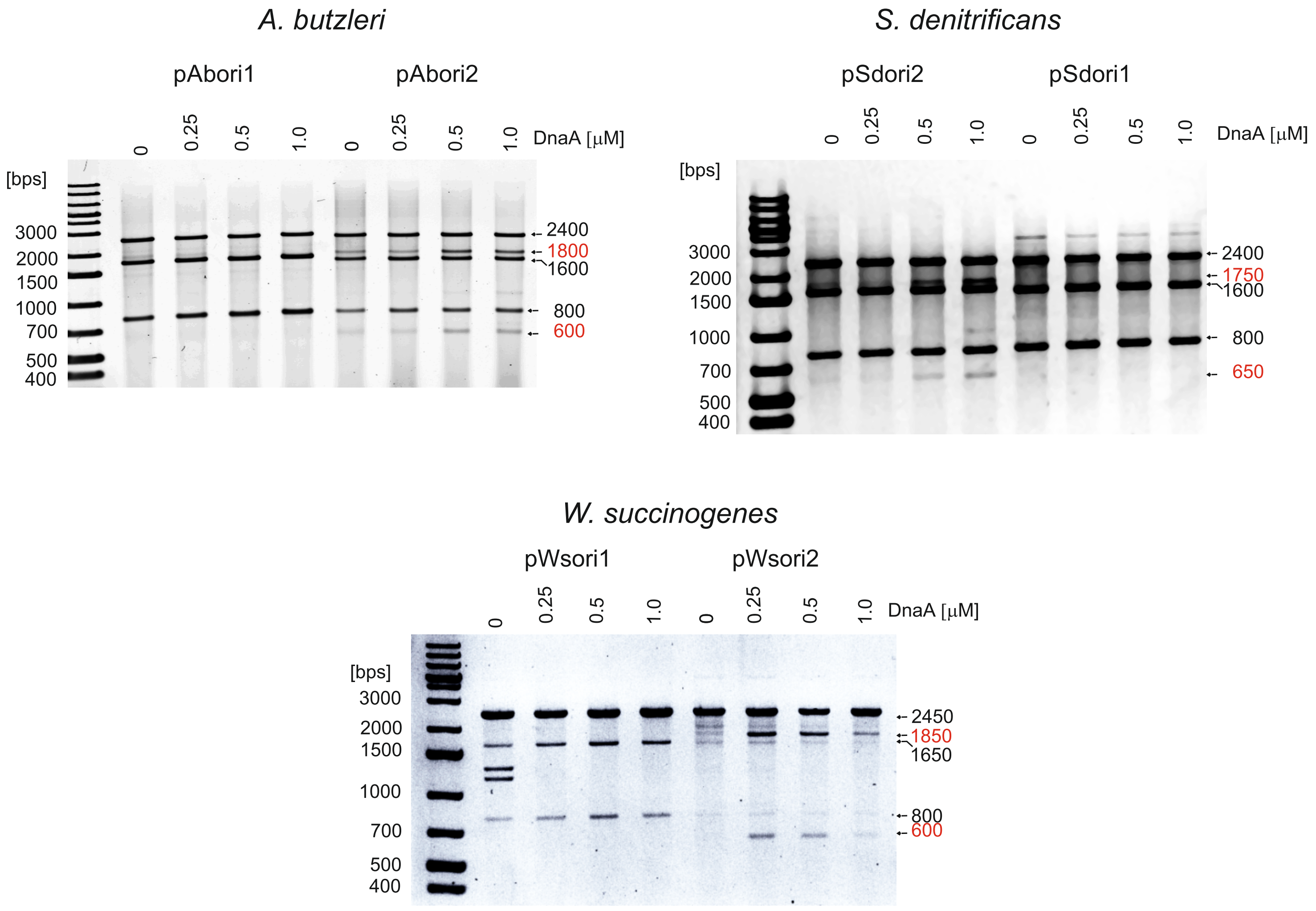

Figure S4. *In vitro* identification of DUEs in putative *oriC* regions of selected Epsilonproteobacteria. P1 nuclease assay determining the DNA region susceptible to DnaA-dependent unwinding. Plasmids containing putative *oriC*s (pAbori1 and pAbori2, pSdori1 and pSdori2, pWsori1 and pWsori2) were incubated with the indicated amounts of species-specific DnaA protein, digested by P1 nuclease, and restriction digested by PvuI or DrdI. The DNA fragments were visualized by separation on 1% agarose gels and ethidium bromide staining. The DNA fragments produced in a DnaA-dependent manner are marked in red. For a plasmid map please refer to the Figure 1.
