# Supplementary material for: Unique and Universal Features of Epsilonproteobacterial Origins of Chromosome Replication and DnaA-DnaA Box Interactions
Source: Front Microbiol. 2016 Sep 30;7:1555. doi: 10.3389/fmicb.2016.01555 (PMC5043019; doi:10.3389/fmicb.2016.01555)
Supplement: Supplementary file 5 [file Image5.PDF]

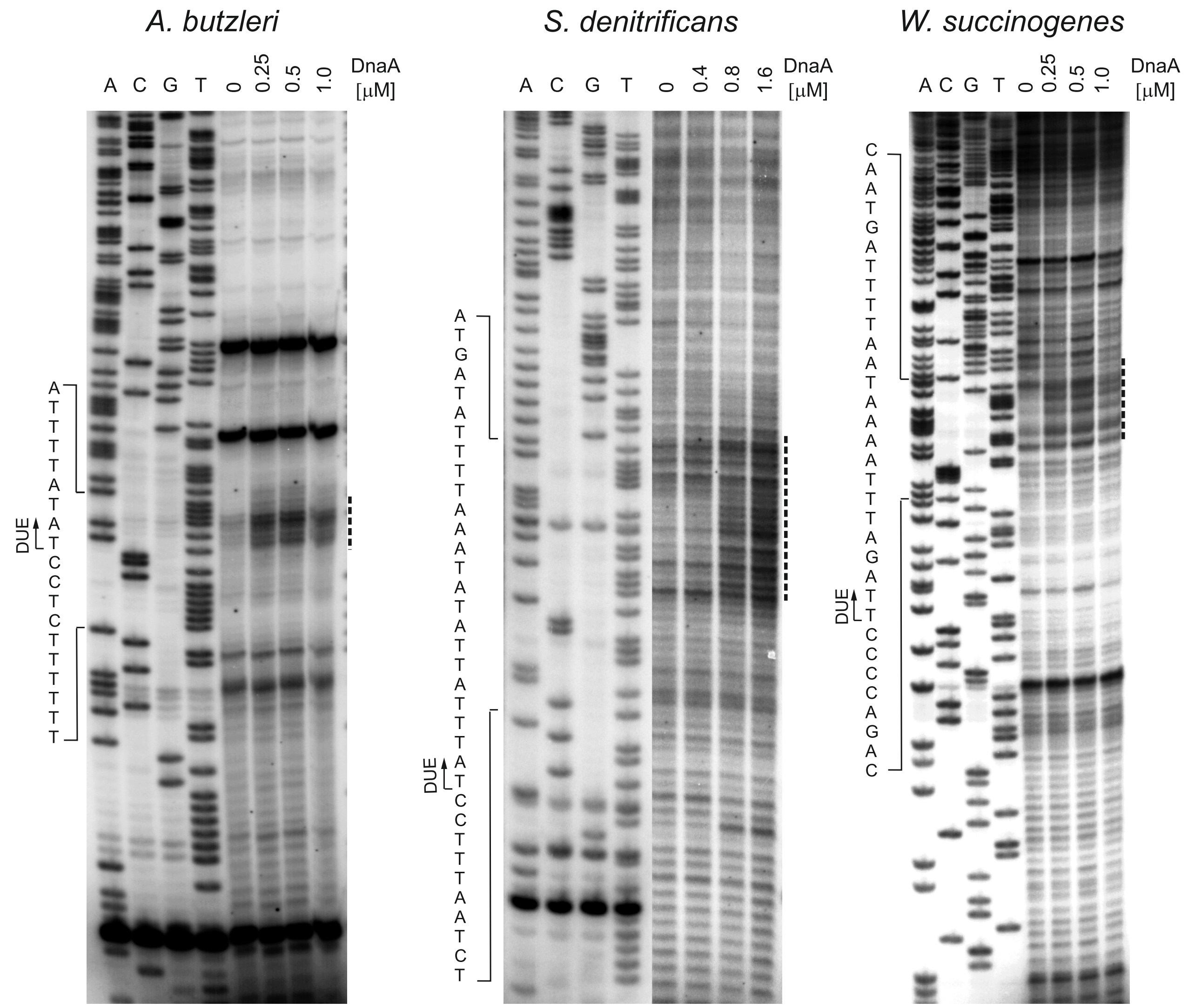

Figure S5. *In vitro* identification of the *A. butzleri, S. denitrificans* and *W. succinogenes oriC* sequence unwound by cognate DnaA proteins. Plasmids: pAbori2*,* pSdori2 and pWsori2, after incubation with the indicated amounts of DnaA, were digested by P1 nuclease and used as templates for PE reactions. Dashed-lines indicate the nucleotides susceptible to P1 nuclease treatment. The boundaries of the DUE are marked with continuous-line arrows next to the presented sequences.
