# Supplementary material for: Unique and Universal Features of Epsilonproteobacterial Origins of Chromosome Replication and DnaA-DnaA Box Interactions
Source: Front Microbiol. 2016 Sep 30;7:1555. doi: 10.3389/fmicb.2016.01555 (PMC5043019; doi:10.3389/fmicb.2016.01555)
Supplement: Supplementary file 6 [file Image6.PDF]

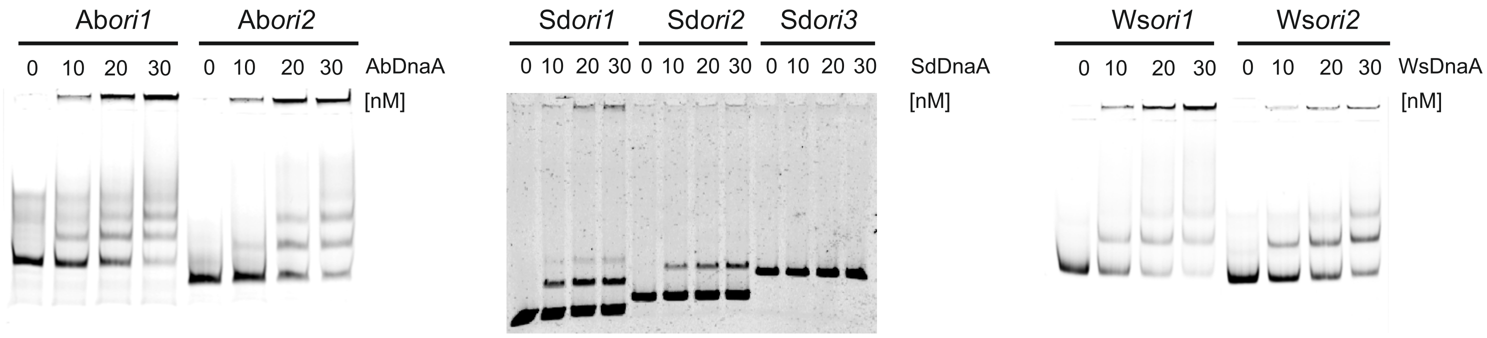

Figure S6. Interaction of DnaA proteins with *oriC* regions. Gel-retardation assay was performed using *oriC* fragments (IRD labelled Ab*ori1*, Ab*ori2*, Ws*ori1*, Ws*ori2* and FAM labelled Sd*ori1*, Sd*ori2* and Sd*ori3*) that were incubated with the increasing amounts of the *A. butzleri* (AbDnaA), *S. denitrificans* (SdDnaA) or *W. succinogenes* (WsDnaA) DnaA proteins. The DNA–protein complexes were separated on a 4 % polyacrylamide gel.
