# Supplementary material for: Unique and Universal Features of Epsilonproteobacterial Origins of Chromosome Replication and DnaA-DnaA Box Interactions
Source: Front Microbiol. 2016 Sep 30;7:1555. doi: 10.3389/fmicb.2016.01555 (PMC5043019; doi:10.3389/fmicb.2016.01555)
Supplement: Supplementary file 7 [file Image7.PDF]

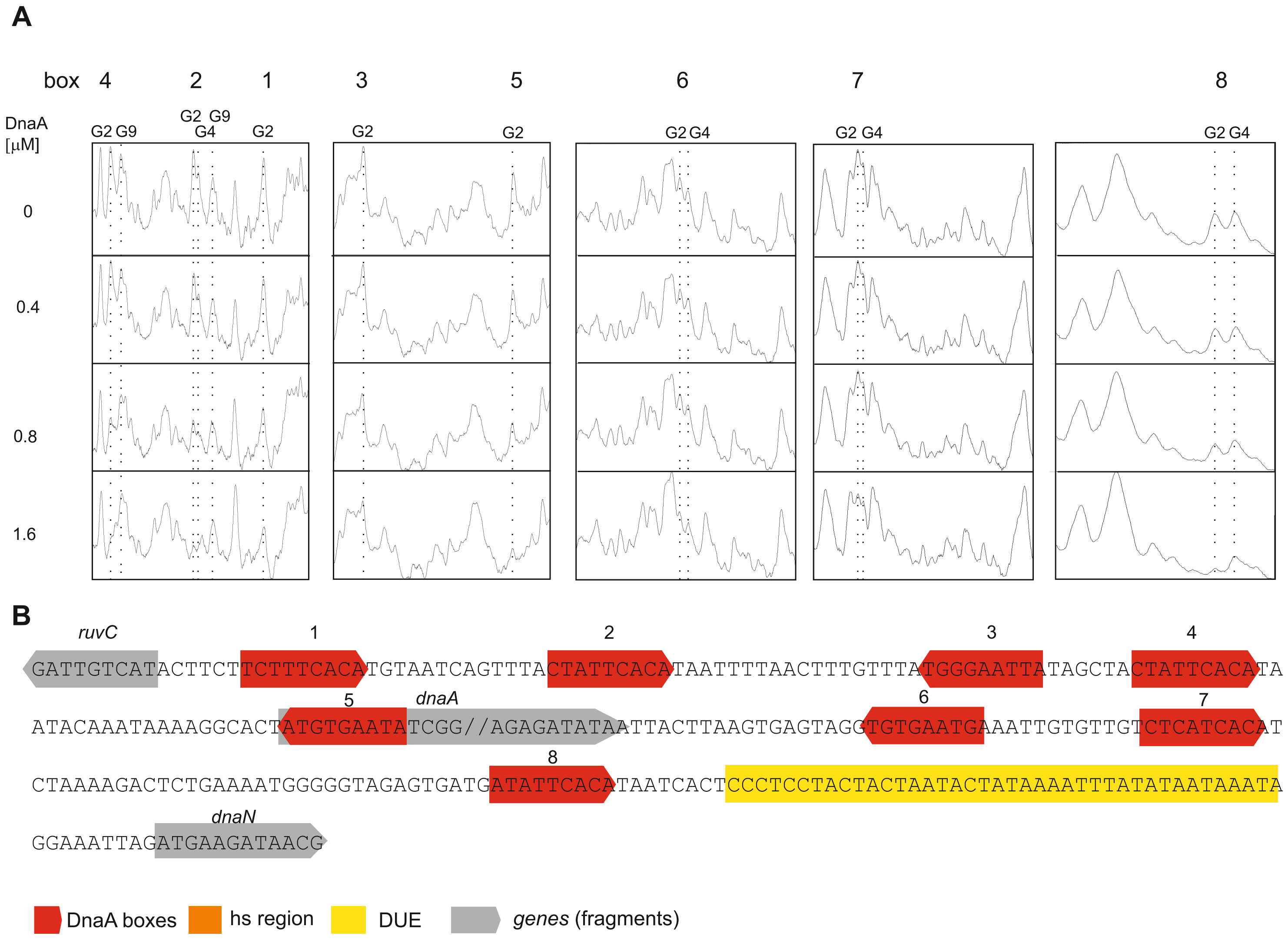

Figure S7. Identification of the DnaA boxes in the *S. denitrificans oriC* region. **(A)** Densitometric plots, which supplement the footprinting data presented on Figure 4. The plots were obtained for the lanes corresponding to the indicated amounts of DnaA protein. Protected guanosine residues (G) are indicated with dotted lines. **(B)** The overall structure of *S. denitrificans oriC* created on the basis of DMS footprint.
