# Supplementary material for: Unique and Universal Features of Epsilonproteobacterial Origins of Chromosome Replication and DnaA-DnaA Box Interactions
Source: Front Microbiol. 2016 Sep 30;7:1555. doi: 10.3389/fmicb.2016.01555 (PMC5043019; doi:10.3389/fmicb.2016.01555)
Supplement: Supplementary file 9 [file Image9.PDF]

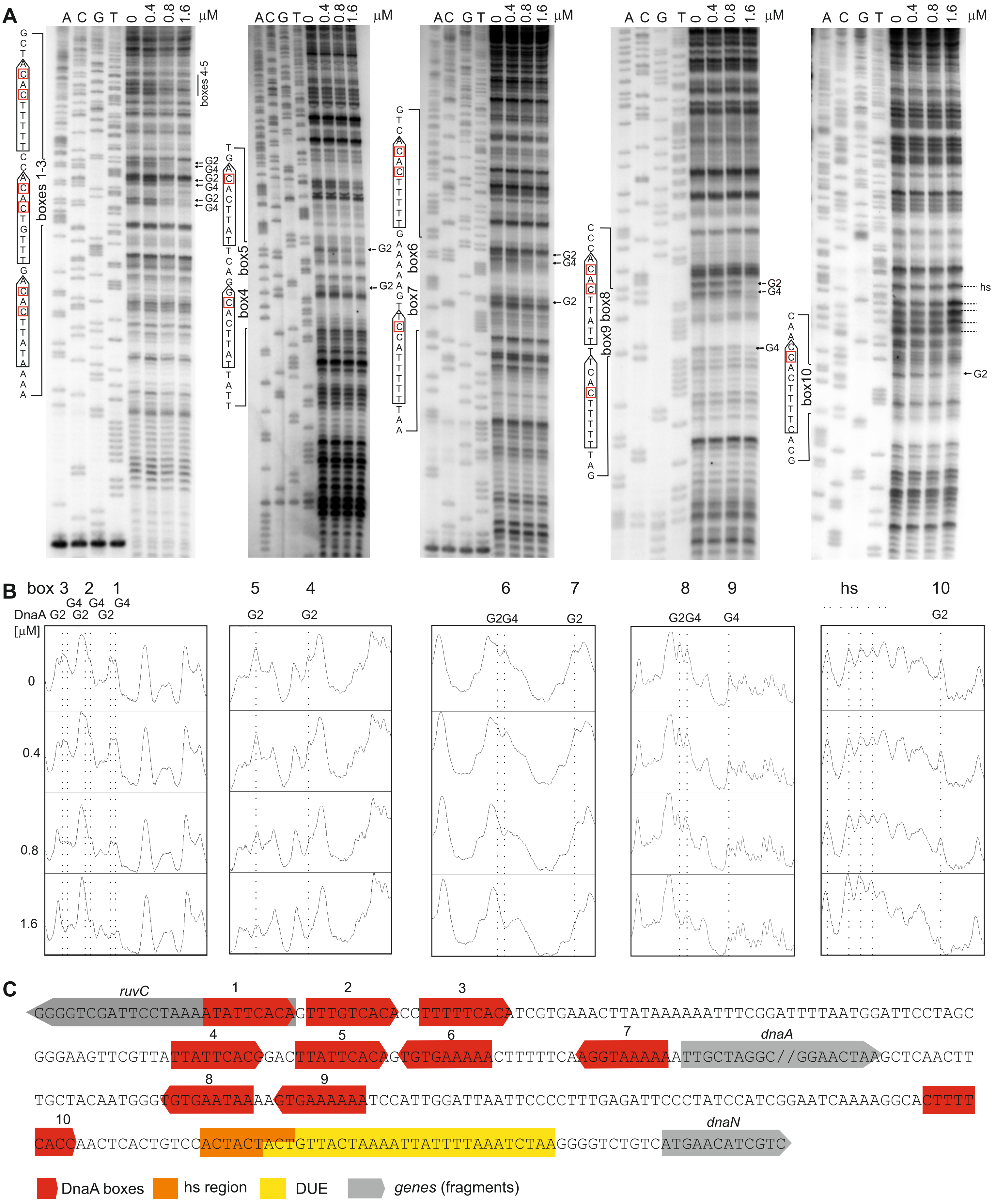

Figure S9. Identification of the DnaA boxes in the *W. succinogenes oriC* region. **(A)** DMS footprint analysis of DnaA-*oriC* interactions. Plasmid pWsori1ori2 was incubated with the indicated DnaA protein concentrations, methylated with DMS and used as a template for PE reactions. Sequences of identified boxes are presented on the left of each panel; arrows and dashed-lines on the right correspond to nucleotides protected from DMS methylation and residues exposed to DMS modification upon protein binding, respectively. **(B)** Densitometric plots, which supplement the footprinting data. The plots were obtained for the lanes corresponding to the indicated amounts of DnaA protein. Protected guanosine residues (G) are indicated with dotted lines. **(C)** The overall structure of *W. succinogenes oriC* created on the basis of DMS footprint.
