# Supplementary material for: Unique and Universal Features of Epsilonproteobacterial Origins of Chromosome Replication and DnaA-DnaA Box Interactions
Source: Front Microbiol. 2016 Sep 30;7:1555. doi: 10.3389/fmicb.2016.01555 (PMC5043019; doi:10.3389/fmicb.2016.01555)
Supplement: Supplementary file 10 [file Image10.PDF]

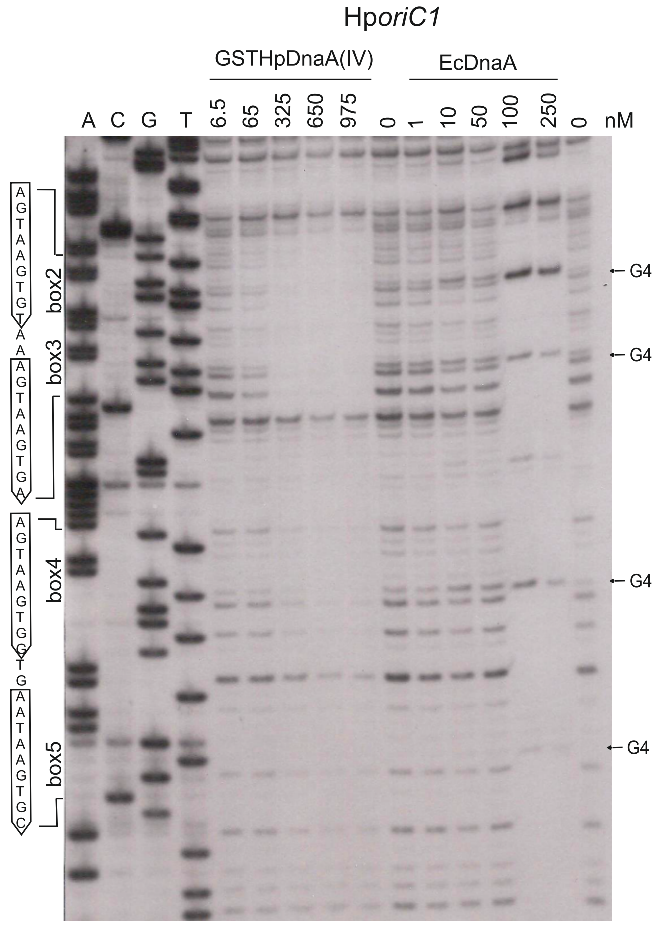

Figure S10. Interaction of the *H. pylori* DnaA (GST-HpDnaA(IV)) protein and the *E. coli* DnaA protein with DNA. DNaseI footprinting analysis was performed using the 187 bp *H. pylori oriC1* that was amplified by ^{32}P labelled P10 and P11 primers. DNA fragments were incubated with the indicated DnaA protein concentrations and treated with DNaseI. Sequences of *H. pylori* DnaA boxes are presented on the left of each panel; arrows on the right correspond to G4 residues.
