# Supplementary material for: Unique and Universal Features of Epsilonproteobacterial Origins of Chromosome Replication and DnaA-DnaA Box Interactions
Source: Front Microbiol. 2016 Sep 30;7:1555. doi: 10.3389/fmicb.2016.01555 (PMC5043019; doi:10.3389/fmicb.2016.01555)
Supplement: Supplementary file 11 [file Image11.PDF]

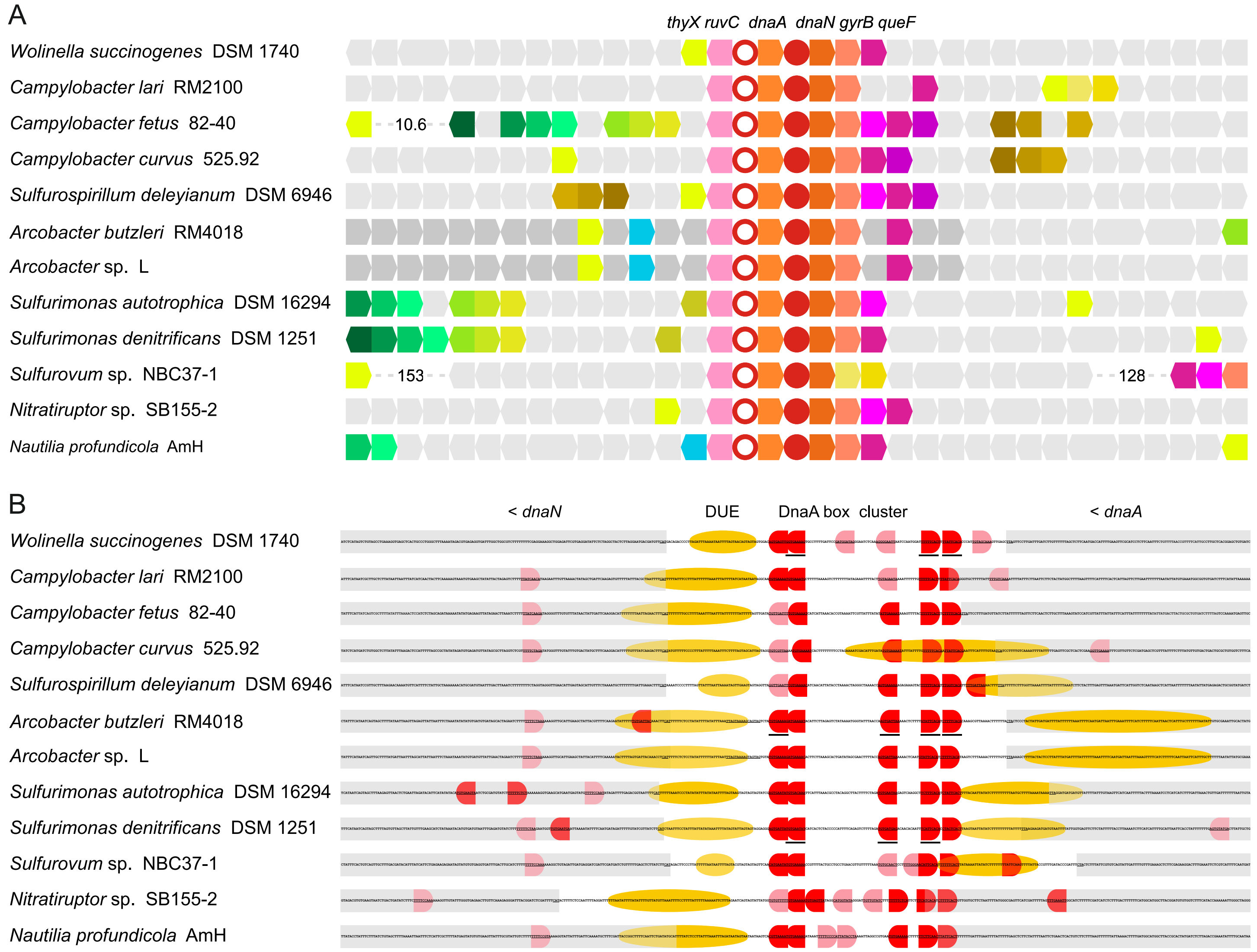

Figure S11. (A) Gene context synopsis of the *oriC* region (+/– 20 kb) for selected Epsilonproteobacteria, including *A. butzleri*, *W. succinogenes*, and *S. denitrificans* analyzed experimentally in this study. Open red circles: *oriC1*; red circles: *oriC2*. Individual genes are shown as boxes, irrespective of gene size, and with arrowheads indicating the direction of their transcription. Blast-homologs are shown in identical colors with the exception of a larger stretch of *A. butzleri* and *Arcobacter* sp. L (dark grey) genes sharing >80% identity to avoid color cluttering. Light grey color indicates genes that have no homologs in any of the other genomes within the chosen ~40 kb window; orthologs may be present in other genome regions, though. The size of three gaps is given in kb. For the accession numbers of the genomes see **B**.

(B) Predictions of *oriC2* structures for selected Epsilonproteobacteria, including *A. butzleri, W. succinogenes,* and *S. denitrificans* analyzed experimentally in this study. Predictions were performed as described in the Materials and Methods section; DnaA boxes (red and pink half-circles) were assigned for sequences matching the *E. coli* consensus (5'-TTWTNCACA, allowing 2 mismatches and 3 mismatches for closely-spaced DnaA boxes); Epsilonproteobacterial-type DnaA boxes (T at the 5th position of a DnaA box) are red while *E. coli* type DnaA boxes (A,C or G at the 5th position of a DnaA box) are pink; DnaA boxes confirmed by experiments in this study are underlined. The sequences were aligned on the positions of their DUE-proximal DnaA box. Strong SIDDs (orange) and weak SIDDs (pale orange) are indicated by ovals; DUE: DNA unwinding element. Open reading frames are indicated by grey boxes; the direction of transcription of the *oriC2*-flanking genes is indicated by arrowheads. *Wolinella succinogenes* DSM 1740 [BX571657.1] rev/compl. pos. 1201..1625; *Campylobacter lari* RM2100 [CP000932.1] rev/compl. pos. 1189..1613; *Campylobacter fetus* subsp. fetus 8For 2-40 [CP000487.1] rev/compl. pos. 1177..1601; *Campylobacter curvus* 525.92 [CP000767.1] rev/compl. pos. 1193..1617; *Sulfurospirillum deleyianum* DSM 6946 [CP001816.1] rev/compl. pos. 2485..2909; *Arcobacter butzleri* RM4018 [CP000361.1] rev/compl. pos. 1204..1628; *Arcobacter* sp. L [AP012048.1] rev/compl. pos. 1214..1638; *Sulfurimonas autotrophica* DSM 16294 [CP002205.1] rev/compl. pos. 1325..1749; *Sulfurimonas denitrificans* DSM 1251 [CP000153.1] rev/compl. pos. 1216..1640; *Sulfurovum* sp. NBC37-1 [AP009179.1] rev/compl. pos. 1313..1737; *Nitratiruptor* sp. SB155-2 [AP009178.1] rev/compl. pos. 1319..1743; *Nautilia profundicola* AmH [CP001279.1] rev/compl. pos. 1266..1690.
