# Supplementary material for: Unique and Universal Features of Epsilonproteobacterial Origins of Chromosome Replication and DnaA-DnaA Box Interactions
Source: Front Microbiol. 2016 Sep 30;7:1555. doi: 10.3389/fmicb.2016.01555 (PMC5043019; doi:10.3389/fmicb.2016.01555)
Supplement: Supplementary file 12 [file Presentation1.PDF]

SUPPLEMENTARY MATERIALS

Unique and universal features of Epsilonproteobacterial origins of chromosome replication and DnaA-DnaA box interactions

Pawel Jaworski, Rafal Donczew, Thorsten Mielke, Marcel Thiel, Stanislaw Oldziej, Christoph Weigel, Anna Zawilak-Pawlik

SUPPLEMENTARY TABLES

Table S1. Strains, plasmids and proteins used in this work.

**Strain/plasmid/ protein**	**Genotype/feature**	**Reference/ source**
**Strains**		
E. coli		
DH5α	F2, Φ80 dlacZΔM15, recA1, endA1, gyrA96, thi-1, hsd R17, (rk - ,mk +), sup E44, relA1, deoR, Δ(lacZYAargF)U169	Laboratory stock
BL21	B F- dcm ompT hsdS(rB- mB)gal	GE Healthcare
**Plasmids**		
pOC170	Cloning plasmid carrying the *E. coli oriC* sequence, the replication origin of pBR322 and the *bla* gene of pBR322	(Messer et al., 1992)
pori1ori2	A pOC170 derivative, lacking *E. coli oriC,* containing DNA fragment of *H.pylori* 26695 genome encompassing *oriC1* and *oriC2* subregions and the *dnaA* gene.	(Donczew et al., 2012)
pAbori1	A pOC170 derivative, lacking *E. coli oriC,* containing *A. butzleri oriC1* region amplified with primers C1 and C2 and cloned between EcoRI and PstI sites	This work
pAbori2	A pOC170 derivative, lacking *E. coli oriC,* containing *A. butzleri oriC2* region amplified with primers C3 and C4 and cloned between EcoRI and PstI sites	This work
pAbori1ori2	A pOC170 derivative, lacking *E. coli oriC,* containing *A. butzleri oriC1-dnaA-oriC2* region amplified with primers C1 and C4 and cloned between EcoRI and PstI sites	This work
pET28AbDnaA	A pET28a derivative containing *A. butzleri dnaA* gene amplified with primers D1 and D2 and cloned between BamHI and XhoI sites	This work
pSdori1	A pOC170 derivative, lacking *E. coli oriC,* containing *S. denitrificans oriC1* region amplified with primers C5 and C6 and cloned between EcoRI and PstI sites	This work
pSdori2	A pOC170 derivative, lacking *E. coli oriC,* containing *S. denitrificans oriC2* region amplified with primers C7 and C8 and cloned between EcoRI and PstI sites	This work
pSdori1ori2	A pOC170 derivative, lacking *E. coli oriC,* containing *S. denitrificans oriC1-dnaA-oriC2* region amplified with primers C5 and C8 and cloned between EcoRI and PstI sites	This work

pET28SdDnaA	A pET28a derivative containing *S. denitrificans dnaA* gene amplified with primers D3 and D4 and cloned between BamHI and XhoI sites	This work
pWsori1	A pOC170 derivative, lacking *E. coli oriC,* containing *W. succinogenes oriC1* region amplified with primers C9 and C10 and cloned between EcoRI and PstI sites	This work
pWsori2	A pOC170 derivative, lacking *E. coli oriC,* containing *W.succinogenes oriC2* region amplified with primers C11 and C12 and cloned between EcoRI and PstI sites	This work
pWsori1ori2	A pOC170 derivative, lacking *E. coli oriC,* containing *W. succinogenes oriC1-dnaA-oriC2* region amplified with primers C9 and C12 and cloned between EcoRI and PstI sites	This work
pET28WsDnaA	A pET28a derivative containing *W. succinogenes dnaA* gene amplified with primers D5 and D6 and cloned between BamHI and XhoI sites	This work
**Proteins**		
6HisAbDnaA	Recombinant, His-tagged *A. butzleri* DnaA protein	This work
6HisSdDnaA	Recombinant, His-tagged *S. denitrificans* DnaA protein	This work
6HisWsDnaA	Recombinant, His-tagged *W. succinogenes* DnaA protein	This work
HpDnaA	Recombinant, untagged *H. pylori* DnaA protein	(Zawilak et al., 2003; Zawilak-Pawlik et al., 2011)
GST-HpDnaA(IV)	Recombinant, GST-tagged domain IV of *H. pylori* DnaA protein	(Zawilak et al., 2001)
EcDnaA	Recombinant, untagged *E. coli* DnaA protein	(Krause and Messer, 1999)

Table S2. Primers used in this work

Name	5’ – 3’ sequence	application
**pOC plasmids**		
C1	CGGAATTCCCATTTTTTTCAACAATCGCATATC	See Materials and Methods
C2	AACTGCAGCTAATTGTTTTAAGTATCTTTCG	
C3	CGGAATTCGTTAATTGAAAAAGATGAAAATTTC	
C4	AACTGCAGAAAAAGGTTGCATTGAAGCTATTAC	
C5	CGGAATTCCTAAAGAGATAAGAGCATAACCC	
C6	AACTGCAGCTATTATATTCTATCTCAGGAATTTC	
C7	CGGAATTCGATTTTAAGGTAAAAATAGAAGAAC	
C8	AACTGCAGGGTTGTGAATGAATTAAAATATTTTC	
C9	CGGAATTCGTGCGCTCTTTGATTTTGATGAG	
C10	AACTGCAGGCTTAATATAGCGCTCATATTC	
C11	CGGAATTCGGATGCGAACTTCAAAATGGTC	
C12	AACTGCAGGTGATTTGGCTGGCGTCTTTTTTTTC	
**pET plasmids**		
D1	CGGGATCCATGACAACTAAAGAGTTTTTAAC	See Materials and Methods
D2	CCGGTCGACCTACTCCCTACTATTGATGATTTTA	

D3	CGGGATCCATGAATATCGGAGAGAAAATTTTAC	
D4	CCGGTCGACTTAAGAAGATGATGTAATTTTATTG	
D5	CGGGATCCATGCTAGGCGATACTACATTAAAAC	
D6	CCGGTCGACTTAGTTCCTTGATTTGATCTTG	
**Primer extension and DnaseI footprinting**		
P1	TCTTTATTCATAATTTTTATTCACA	Figure S8, boxes 3 and 4
P2	AGAGTTTTTAATCACTTGGTTAAAT	Figures 2 and S8, box 8
P3	TGTGAAAAGATGAAAATACATTCTT	Figure S8, boxes 5 and 6
P4	TTGTCACACCTTTTTCACATCGT	Figure S9, boxes 4 and 5
P5	CAATGGGTGTGAATAAAAGTGAA	Figures 2 and S9, box 10
P6	GTGCCTTTTGATTCCGATGGA	Figure S9, boxes 8 and 9
P7	AGTAGGAGGGAGTGATTATGTGA	Figure 4, box 6
P8	AAGTGAGTAGGTGTGAATGAAA	Figures 2, and 4, box 8
P10	GCAAAGCAGCATGAAAATC	Figure S10
P11	CAATATTGTTGTTGGTATCC	Figure S10
P12	CCCTTAAAAGAAACACCTTAA	Figure 6, boxes 2,3,4 and 5
P13	GTTTTTGAGTTGTGTATAACCCCT	Figure 6, boxes R1 and R5
C1	CGGAATTCCCATTTTTTTCAACAATCGCATATC	Figure S8, boxes 1 and 2
C3	CGGAATTCGTTAATTGAAAAAGATGAAAATTTC	Figure S8, box 7
C5	CGGAATTCCTAAAGAGATAAGAGCATAACCC	Figure 4, boxes 1,2 and 4
C6	AACTGCAGCTATTATATTCTATCTCAGGAATTTC	Figure 4, boxes 3 and 5
C7	CGGAATTCGATTTTAAGGTAAAAATAGAAGAAC	Figure 4, box 7
C9	CGGAATTCGTGCGCTCTTTGATTTTGATGAG	Figure S9, boxes 1,2 and 3
C10	AACTGCAGGCTTAATATAGCGCTCATATTC	Figure S9, boxes 6 and 7
**EMSA**		
E1	IRD800-GGAGTAAGAATAGCTTCGAAT	Figure S6
E2	IRD800-CATCGATAGGATATCCTGGG	Figure S6
E3	FAM-GGAGTAAGAATAGCTTCGAAT	Figure S6
E4	FAM-CATCGATAGGATATCCTGGG	Figure S6

SUPPLEMENTARY METHODS

In silico origin predictions

The prediction of *oriC*-type replication origins in the genomes of *A. butzleri* RM4018 [GenBank entry CP000361.1], *S. denitrificans* DSM 1251 [GenBank entry CP000153.1.1], *W. succinogenes* DSM 1740 [GenBank entry BX571656.1], and *H. hepaticus* ATCC 51449 [GenBank entry AE017125.1] was performed in a stepwise procedure. First, the annotation of the *dnaA* gene in the genome was validated by TBLASTN (version 2.2.30) (Shiryev et al., 2007) using the DnaA sequence of *H. pylori* 26695 [GenBank entry AAD08568.1] as query. Second, the approximate *oriC* location in the genome was determined using as a guide the inflection point (minimum) of its cumulative GC-skew provided by the Comparative Genometrics website (Roten et al., 2002) or obtained from the GenSkew webserver (http://genskew.csb.univie.ac.at/); Third, WebSIDD (Bi and Benham, 2004) (http://benham.genomecenter.ucdavis.edu/sibz/) was used with default settings (37°C, 0.1 M salt, circular DNA, copolymeric) and negative superhelicity values in the range of $\sigma = -0.04$ (low) to $\sigma = -0.06$ (high) in increments of 0.005 for the prediction of putative DUE(s) (Kowalski and Eddy, 1989) in intergenic regions in the vicinity (+/– 10 kb) of the GC-skew inflection point (minimum). Fourth, DnaA boxes were assigned manually using the *E. coli* consensus 5'-TTWTNCACA (Schaper and Messer, 1995) and allowing for 2 mismatches (3 mismatches for

closely-spaced DnaA boxes). Finally, a prediction was considered significant if one DnaA box in reverse orientation (5'-TGTGNAWAA) could be assigned to a position of ~2 helical turns distant to the border of a strong DUE. We derived this latter criterion by comparison of the distances and orientations of the DUE-proximal DnaA boxes in *oriC* predictions for *E. coli* K-12, *B. subtilis* 168, and *H. pylori* 26695 with the experimentally found unwinding points in the respective *oriC*s (Donczew et al., 2012; Krause and Messer, 1999). Prediction output data were obtained as raw text files and further processed with Microsoft Excel v97SR-1 and Corel Draw v.11.

Protein expression and purification

The *dnaA* genes were amplified by PCR using primer pairs: D1-D2, D3-D4, D5-D6 and inserted between the BamHI-XhoI restriction sites of pET28a(+) to give pET28AbDnaA, pET28SdDnaA and pET28WsDnaA, respectively. Proteins were expressed and purified as described previously (Zawilak-Pawlik et al., 2006). Briefly, LB medium (800 ml) was inoculated 1:50 with an overnight culture of *E. coli* containing the proper expression vector. The cells were grown at 37°C until the optical density (OD_{600}) reached 1.8 and subsequently induced with IPTG to give a final concentration of 0.5 mM. The growth was continued for 3 h at 30°C, and the culture was harvested by centrifugation (10 min, 5,000 g, 4°C). The bacterial pellet was stored at -20°C until purification. The thawed cells were suspended in 40 ml of ice-cold buffer L (25 mM Hepes/KOH, pH 7.6, 100 mM potassium glutamate, 1 mM DTT) complemented with SIGMA FAST Protease Inhibitor Cocktail (Sigma) and lysozyme (Fluka) at a final concentration of 1 mg/ml and incubated on ice for 30 min. Then, the cells were disrupted by sonication and centrifuged (40 min, 27,000 g, 4°C). The supernatant was decanted. Subsequently, the proteins were precipitated with ammonium sulphate (0.34 g/ml) and centrifuged (30 min, 27,000 g, 4°C). The protein pellet was stored at -20°C until the next purification steps. The protein pellet was thawed on ice and suspended in 12 ml of ice-cold LG_{100} buffer (45 mM Hepes/KOH, pH 7.6, 100 mM potassium glutamate, 10 mM magnesium acetate, 1 mM DTT, 0.5 mM EDTA and 20% sucrose). The mixture was applied to PD-10 columns (GE Healthcare) and eluted with LG_{100} buffer. The proteins were diluted to 15 ml with LG_{100} buffer, and Ni^{2+}-NTA agarose was added (0.5 ml bed volume). The resin was incubated with the proteins for 1 h on ice under gentle agitation. The agarose beads were centrifuged (5 min, 500 g, 4°C), washed three times with 15 ml LG_{100} buffer supplemented with 10 mM imidazole and transferred to the column. The elution was carried out with LG_{100} buffer supplemented with 20 mM imidazole (3 x 0.35 ml), with 50 mM imidazole (4 x 0.35 ml) and finally with 100 mM imidazole (4 x 0.35 ml). In all subsequent analyses DnaA was supplemented with 3 mM ATP (EM) or 5 mM ATP (footprinting and P1 nuclease assay).

DMS footprinting

In vitro DNA modification was performed as described previously (Donczew et al., 2014; Sasse-Dwight and Gralla, 1991). Mixtures in a total volume of 50 µL FB buffer (25 mM Hepes-KOH (pH 7.6), 12% (v/v) glycerol, 1 mM $CaCl_2$, 0.2 mM EDTA, 5 mM ATP, 0.1 mg/ml BSA) containing 1 µg of plasmid DNA (approximately 10 nM) and appropriate DnaA protein (0.2, 0.4 and 0.8 µM or 0.4, 0.8, 1.6 µM) were incubated at 30°C for 10 min. Subsequently, 3.3 µL of 150 mM DMS (Sigma-Aldrich) was added to a final concentration of 9.3 mM, and the incubation was continued for 5 min. The modification reaction was inhibited by the addition of 100 µL cold Stop Buffer (3 M ammonium acetate, 1 M 2-mercaptoethanol and 20 mM EDTA) and a short incubation on ice. Modified plasmids were precipitated with 300 µL of cold ethanol, dried, resuspended in 100 µL of 1 M piperidine and incubated for 30 min at 90°C. Fragmented DNA was purified by gel filtration on Sephacryl S-500 (Sigma-Aldrich) spin columns

equilibrated in molecular-grade water. Modifications were followed by primer extension (PE) analyses.

DNaseI footprinting

DNAseI footprinting on linear DNA was performed as described previously (Roth et al., 1994; Zawilak et al., 2001). *H. pylori oriC1* and *E. coli oriC* were amplified by PCR using HPS1a-HPS1b and Ecorifw-Ecorirev primers; HpS1b and Ecorifw were labelled at 5' with $^{[32]}$P. DNaseI footprinting was performed in a total volume of 20 µL FDB buffer (FB buffer supplemented with 5 mM $CaCl_2$ and 10mM $MgCl_2$) containing approximately 100 fmol of DNA and appropriate DnaA protein (6.5-2600 nM GST-HpDnaA(IV) or 1-250 nM EcDnaA) were incubated at 37°C for 30 min. Subsequently, 2-5 µL of DNaseI (0,125 pg/ml mg/ml) was added, and the incubation was continued for 5 min. The reaction was inhibited by the addition of 30 µL Stop Buffer (1% SDS, 200 mM NaCl, 20 mM EDTA and 40 µg/ml tRNA) and a short incubation on ice. Fragmented DNA was extracted by phenol-chloroform and precipitated with ice-cold ethanol, dried, resuspended in 4 µL of TE (10 mM Tris-HCl, pH 8.0, 1 mM EDTA). The DNAseI cleave products were separated on an 8% polyacrylamide gel under denaturing conditions and visualized with a Typhoon FLA9500 Variable Mode Imager (GE Healthcare).

P1 nuclease assay

The P1 nuclease assay was conducted similarly as described (Donczew et al., 2012). Reaction mixtures (15 µL) of FB buffer containing 300 ng of plasmid DNA (approx. 10 nM) and appropriate 6HisDnaA protein (up to 4 µM) were incubated for 10 min at 30°C followed by P1 nuclease (Sigma-Aldrich) addition (0.45 unit in 0.01 M sodium acetate pH 7.6), and the incubation was continued for 5 min at 30°C. The reaction was stopped by the addition of 85 µL of water and 100 µL of Binding Buffer (GeneJET, Thermo Scientific), and samples were placed on ice. DNA was subsequently purified with GeneJET Gel Extraction Kit columns. The P1 activity was analyzed by restriction enzyme digestion or PE analysis. The first method required the digestion of purified DNA after P1 treatment with PvuI or DrdI and 1% agarose gel separation with subsequent ethidium bromide staining. The gels were scanned with a Typhoon 8600 Variable Mode Imager (GE Healthcare) or GelDoc Xr+ Imaging System (Bio-Rad).

Primer extension (PE) reactions

PE analysis was used to determine the modification sites introduced either by DMS or P1 nuclease. A single PE reaction (30 cycles of 30 s at 95°C, 30 s at 50°C and 60 s at 72°C) was carried out in a mixture containing 0.3 units of Taq polymerase (Thermo Scientific), 25 fmol of DNA template and 1 pmol ^{32}P-labeled primer. After PE, mixtures were separated on an 8% polyacrylamide gel under denaturing conditions and visualized with a Typhoon 8600 Variable Mode Imager (GE Healthcare).

Electrophoretic mobility shift assay (EMSA)

The EMSA was used to identify DnaA protein binding to putative *oriC* subregions. Reaction mixtures (15 µL) containing 75 fmol of IRD800 or FAM-labelled DNA, 20 mM Hepes-KOH (pH 7.6), 5 mM magnesium acetate, 1 mM EDTA, 4 mM DTT, 0.2% Triton X-100, 3 mM ATP, 0,5 g/l BSA, 50 ng of non-specific DNA competitor (P0307; Sigma) and 6HisDnaA protein (up to 30 nM) were incubated for 15 min at room temperature. The bound complexes were separated by electrophoresis in 4% polyacrylamide gels (0.25x or 1x TBE at

7.5 V/cm, 4°C). Results were visualized by an Odyssey CLx Infrared Imaging System (Li-Cor) or Typhoon FLA9500 (GE Healthcare).

Electron microscopy (EM)

Electron microscopy was performed as described previously (Donczew et al., 2012, 2014), with few modifications. 60 ng (approx. 60 nM) of *S. denitrificans* and *W. succinogenes* and 90 ng (approx. 90 nM) of *A. butzleri* DnaA proteins were incubated with 60 ng (approx. 1 nM) of plasmid DNA: pSdori1ori2, pWsori1ori2 and pAbori1ori2, respectively. The images were analyzed by a ImageJ software. To calculate the binding and distribution of complexes approximately 200 DNA molecules were analyzed for each experimental set.

SUPPLEMENTARY REFERENCES

Bi, C., and Benham, C. J. (2004). WebSIDD: server for predicting stress-induced duplex destabilized (SIDD) sites in superhelical DNA. *Bioinforma. Oxf. Engl.* 20, 1477–1479. doi:10.1093/bioinformatics/bth304.

Donczew, R., Mielke, T., Jaworski, P., Zakrzewska-Czerwińska, J., and Zawilak-Pawlik, A. (2014). Assembly of *Helicobacter pylori* initiation complex is determined by sequence-specific and topology-sensitive DnaA-*oriC* interactions. *J. Mol. Biol.* 426, 2769–2782. doi:10.1016/j.jmb.2014.05.018.

Donczew, R., Weigel, C., Lurz, R., Zakrzewska-Czerwinska, J., and Zawilak-Pawlik, A. (2012). *Helicobacter pylori oriC*-the first bipartite origin of chromosome replication in Gram-negative bacteria. *Nucleic Acids Res.* doi:10.1093/nar/gks742.

Kowalski, D., and Eddy, M. J. (1989). The DNA unwinding element: a novel, cis-acting component that facilitates opening of the *Escherichia coli* replication origin. *EMBO J.* 8, 4335–4344.

Krause, M., and Messer, W. (1999). DnaA proteins of *Escherichia coli* and *Bacillus subtilis*: coordinate actions with single-stranded DNA-binding protein and interspecies inhibition during open complex formation at the replication origins. *Gene* 228, 123–132.

Messer, W., Hartmann-Kühlein, H., Langer, U., Mahlow, E., Roth, A., Schaper, S., et al. (1992). The complex for replication initiation of *Escherichia coli*. *Chromosoma* 102, S1-6.

Ozaki, S., Fujimitsu, K., Kurumizaka, H., and Katayama, T. (2006). The DnaA homolog of the hyperthermophilic eubacterium *Thermotoga maritima* forms an open complex with a minimal 149-bp origin region in an ATP-dependent manner. *Genes Cells Devoted Mol. Cell. Mech.* 11, 425–438. doi:10.1111/j.1365-2443.2006.00950.x.

Roten, C.-A. H., Gamba, P., Barblan, J.-L., and Karamata, D. (2002). Comparative Genometrics (CG): a database dedicated to biometric comparisons of whole genomes. *Nucleic Acids Res.* 30, 142–144.

Roth, A., Urmoneit, B., and Messer, W. (1994). Functions of histone-like proteins in the initiation of DNA replication at *oriC* of *Escherichia coli*. *Biochimie* 76, 917–923.

Sasse-Dwight, S., and Gralla, J. D. (1991). Footprinting protein-DNA complexes *in vivo*. *Methods Enzymol.* 208, 146–168.

Schaper, S., and Messer, W. (1995). Interaction of the initiator protein DnaA of *Escherichia coli* with its DNA target. *J. Biol. Chem.* 270, 17622–17626.

Schaper, S., Nardmann, J., Lüder, G., Lurz, R., Speck, C., and Messer, W. (2000). Identification of the chromosomal replication origin from *Thermus thermophilus* and its interaction with the replication initiator DnaA. *J. Mol. Biol.* 299, 655–665. doi:10.1006/jmbi.2000.3764.

Shiryev, S. A., Papadopoulos, J. S., Schäffer, A. A., and Agarwala, R. (2007). Improved BLAST searches using longer words for protein seeding. *Bioinforma. Oxf. Engl.* 23, 2949–2951. doi:10.1093/bioinformatics/btm479.

Watanabe, S., Ohbayashi, R., Shiwa, Y., Noda, A., Kanesaki, Y., Chibazakura, T., et al. (2012). Light-dependent and asynchronous replication of cyanobacterial multi-copy chromosomes. *Mol. Microbiol.* 83, 856–865.

Zawilak, A., Cebrat, S., Mackiewicz, P., Król-Hulewicz, A., Jakimowicz, D., Messer, W., et al. (2001). Identification of a putative chromosomal replication origin from *Helicobacter pylori* and its interaction with the initiator protein DnaA. *Nucleic Acids Res.* 29, 2251–2259.

Zawilak, A., Durrant, M. C., Jakimowicz, P., Backert, S., and Zakrzewska-Czerwińska, J. (2003). DNA binding specificity of the replication initiator protein, DnaA from *Helicobacter pylori. J. Mol. Biol.* 334, 933–947.

Zawilak-Pawlik, A., Donczew, R., Szafrański, S., Mackiewicz, P., Terradot, L., and Zakrzewska-Czerwińska, J. (2011). DiaA/HobA and DnaA: a pair of proteins co-evolved to cooperate during bacterial orisome assembly. *J. Mol. Biol.* 408, 238–251. doi:10.1016/j.jmb.2011.02.045.

Zawilak-Pawlik, A. M., Kois, A., and Zakrzewska-Czerwinska, J. (2006). A simplified method for purification of recombinant soluble DnaA proteins. *Protein Expr. Purif.* 48, 126–133. doi:10.1016/j.pep.2006.01.010.
